# Supplementary material for: Single-Cell RNA-Sequencing Reveals a Continuous Spectrum of Differentiation in Hematopoietic Cells
Source: Cell Rep. 2016 Jan 21;14(4):966–77. doi: 10.1016/j.celrep.2015.12.082 (PMC4742565; doi:10.1016/j.celrep.2015.12.082)
Supplement: Document S1. Supplemental Figures S1–S7 [file mmc1.pdf]

Cell Reports
Supplemental Information

Single-Cell RNA-Sequencing Reveals a Continuous Spectrum of Differentiation in Hematopoietic Cells

Iain C. Macaulay, Valentine Svensson, Charlotte Labalette, Lauren Ferreira, Fiona Hamey, Thierry Voet, Sarah A. Teichmann, and Ana Cvejic

Supplemental Table legends

Table S1, Related to Figure 4. Top genes distinguishing each cluster based on random forest feature importance.

Table S2, Related to Figure 6. List of genes with dynamic expression over pseudotime, annotated by which temporal expression group they belong to. Together with enriched functional gene sets for the different temporal expression groups.

Table S3, Related to Figure 7. List of expressed Ohnolog gene pairs annotated by the decision tree classification into Single, Mixed and XOR -Ohnologs.

Supplemental Figure Legends

Figure S1. The gating strategy for sorting cd41-EGFP cells by flow cytometry, Related to Figure 1. First, debris was excluded by forward and side-scatter (A, D). Next, singlets were selected (B, E) and dead, PI positive cells, were excluded (C, F). Finally, autofluorescent cells were excluded from the analysis (G, H). The GFP positive population was split into GFP^{low} and GFP^{high} based on the level of GFP fluorescence (I).

Figure S2. Quality control assessment, Related to Figure 1. Quality control was assessed by analysing the number of detected genes compared to the number of input reads (A) or ERCC content (B). In each plate we sorted 94 cells, leaving two wells per plate without cells. Blue dots represent wells with cells and orange dots show wells without cells. Following sequencing and quality control, 13 cells were removed from further analysis. We excluded data points (cells) with few reads (less than 50,000) and few genes or with high ERCC content. As expected, wells without cells (orange) have ERCC content equivalent to 100%.

Figure S3. Pairwise plots of the four independent components used to represent the data, Related to Figure 2. A) The initial names of the components ("difference_component", "outlier_component", "within_large_component", "within_small_component") were given based on visual features. The dots, representing cells, are colored white for EGFP^{low} sorted cells and green for EGFP^{high} sorted cells. B) Ward clustering of the cells in ICA space. The clusters (here colored) were used to associate cells to progression along a component where the cluster varies the most.

Figure S4. The gating strategy for sorting cells from clusters 1a/1b/2, 3 and 4 by flow cytometry, Related to Figure 4. A-B) Plots of viable, single cells based on their GFP and PERCP fluorescence from either a non transgenic (A) or Tg(cd41:EGFP) (B) kidney single cell suspension. The GFP^{low} cells (C) can be further split into two groups based on SSC values: GFP^{low}SSC^{high} or GFP^{low}SSC^{low} (D). GFP fluorescence (E) and light scatter (F) properties of each cell coloured based on the cluster it belongs to. G) Stacked column graph showing the proportion of cells from each of the clusters in three different gates named here: GFP^{high}, GFP^{low}SSC^{low} and GFP^{low}SSC^{high}.

Figure S5. May-Grünwald Giemsa staining of cells from clusters 1a/1b/2, 3 and 4, Related to Figure 4. Cd41:EGFP cells were sorted based on GFP and SSC values to GFP^{low}SSC^{high},GFPl^{ow}SSC^{low} and GFP^{high}. Cytospin slides were prepared from sorted cells and stained with May-Grünwald Giemsa. The GFP^{low}SSC^{high} cells are enriched for cells from clusters 1a/1b/2, GFP^{low}SSC^{low} and GFP^{high} cells are enriched for cells from cluster 3 and 4 respectively.

Figure S6. Follow-up experiment, Related to Figure 5. A) Quality control of cells from the follow-up experiment. Out of 288 single cells, 19 were removed from further analysis due to having less than 200,000 sequenced reads, less than 150 detected genes or more than 99.5% ERCC spike-in content in the well. Thresholds were guided by control wells which were either empty or contained 50 cells. B) The data follow a similar pattern as in the original experiment (for comparison please see Figure S3A-B). Pairwise plots of three independent components representing the data from the follow-up experiment. The EarlyEnriched population is confined to the early progression along component 0 (corresponding to within_small_component in Figure S3B) before the switch in component 2 (corresponding to difference_component in Figure S3B). This corresponds to cluster 1a/1b/2 in the original data as expected. GFP^{high} cells from both the kidney and circulation completely overlap, indicating no further differentiation happens after the cells leave the kidney, and vary over component 1 (corresponding to within_large_component in Figure S3B).

Figure S7. The total mRNA content and number of expressed genes per cell are correlated with its differentiation state, not technical properties of the cells, Related to Figure 5. Light scatter properties FSC and SSC, total mRNA content, number of reads and the number of expressed genes in pseudotime. The dots, representing cells, are coloured based on the cluster the cells belong to.

Supplemental Data

Supplemental Data 1. Sample info, Related to Figures 1-7.

This table contains detailed information about each sample, which was inferred by analysis and used to create most figure panels.

Supplemental Data 2. Analysis files, Related to Figures 1-7.

This contains the scripts, in the form of IPython notebooks, to reproduce all analysis and most of the figure panels in the text.

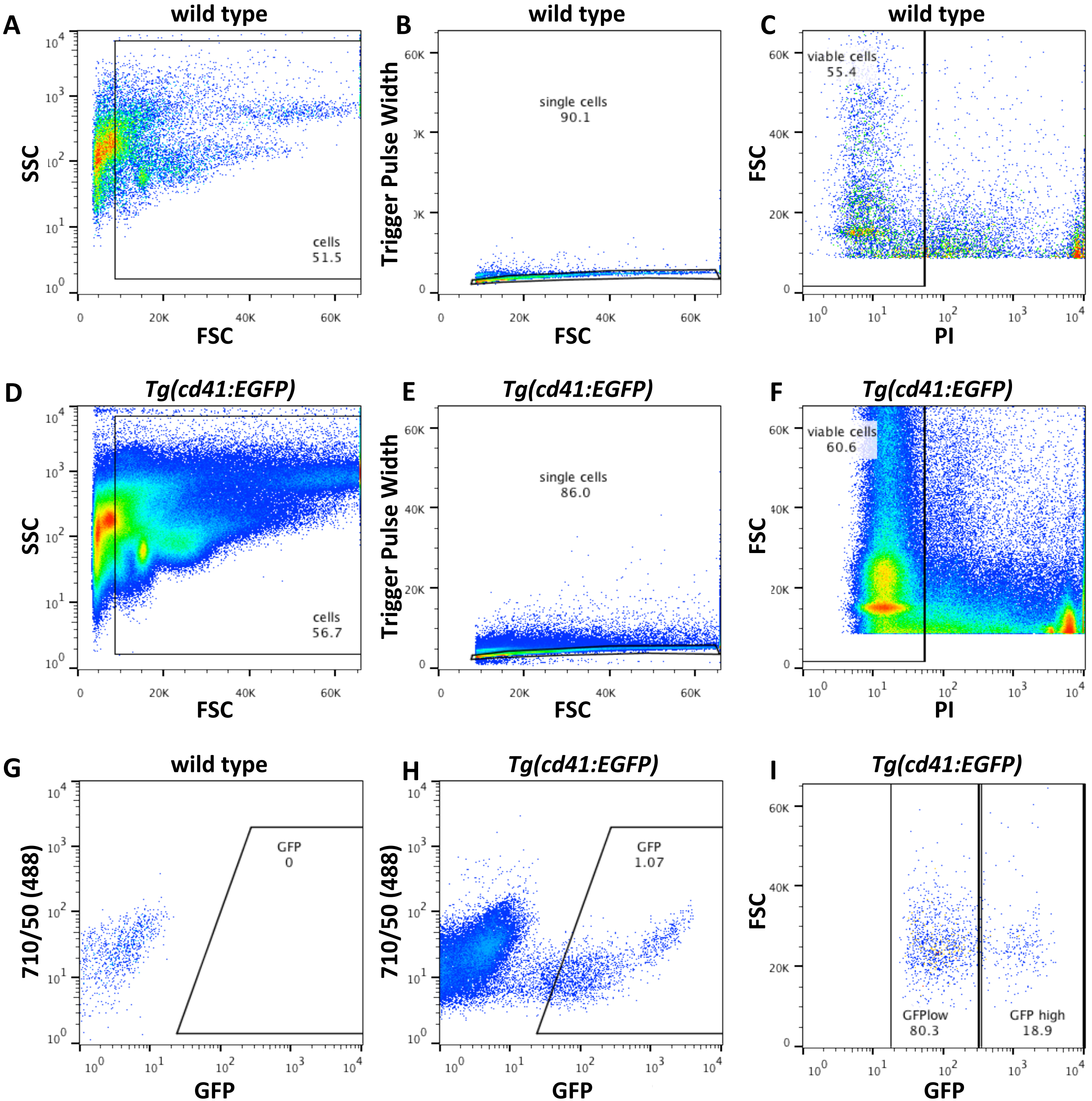

A
wild type
SSC
FSC
cells
51.5
B
wild type
Trigger Pulse Width
FSC
single cells
90.1
C
wild type
viable cells
55.4
FSC
PI
D
Tg(cd41:EGFP)
SSC
FSC
cells
56.7
E
Tg(cd41:EGFP)
Trigger Pulse Width
FSC
single cells
86.0
F
Tg(cd41:EGFP)
viable cells
60.6
FSC
PI
G
wild type
710/50 (488)
GFP
GFP
0
H
Tg(cd41:EGFP)
710/50 (488)
GFP
GFP
1.07
I
Tg(cd41:EGFP)
FSC
GFP
GFPlow
80.3
GFP high
18.9

Figure_S1

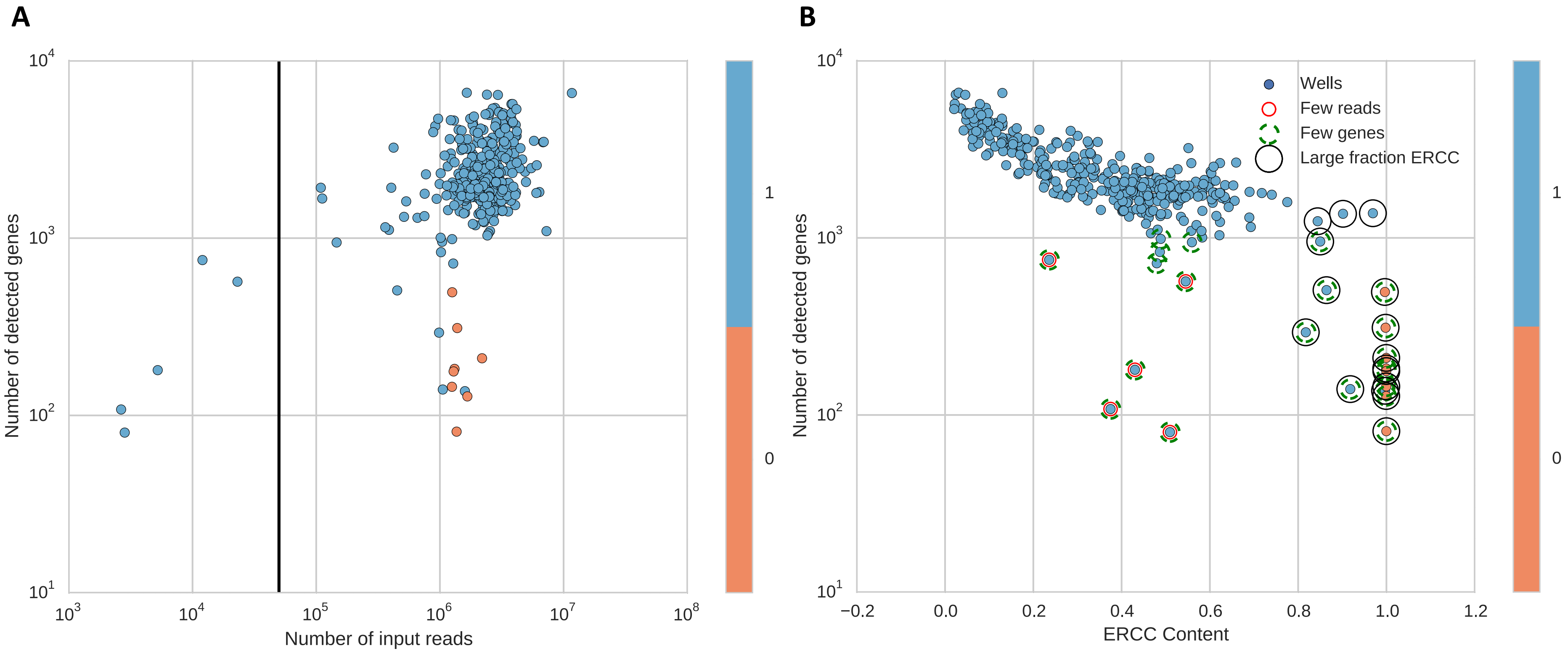
A
B
Number of detected genes
Number of input reads
ERCC Content
Wells
Few reads
Few genes
Large fraction ERCC
1
0

Figure_S2

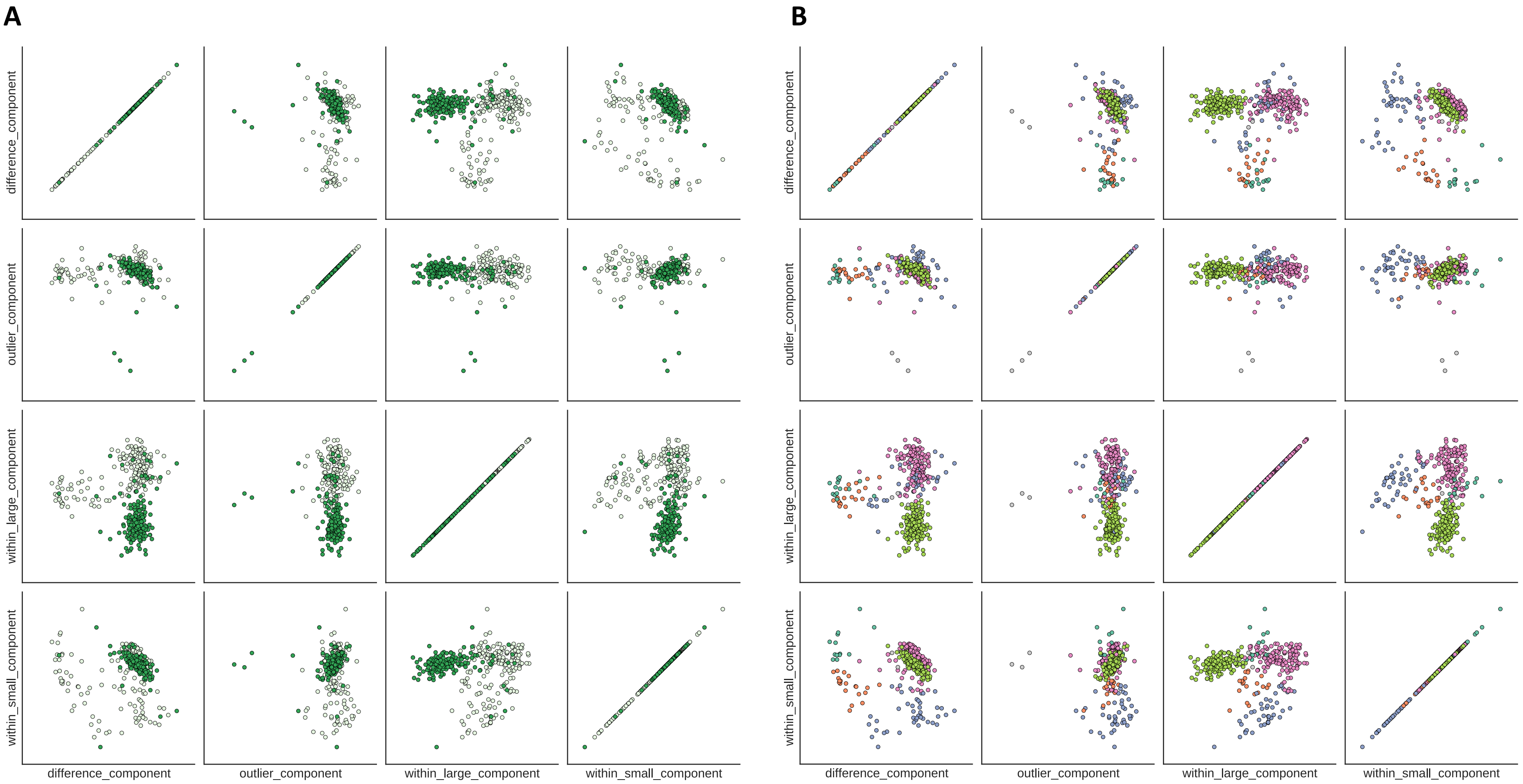
A
B
difference_component
outlier_component
within_large_component
within_small_component
difference_component
outlier_component
within_large_component
within_small_component
difference_component
outlier_component
within_large_component
within_small_component
difference_component
outlier_component
within_large_component
within_small_component

Figure_S3

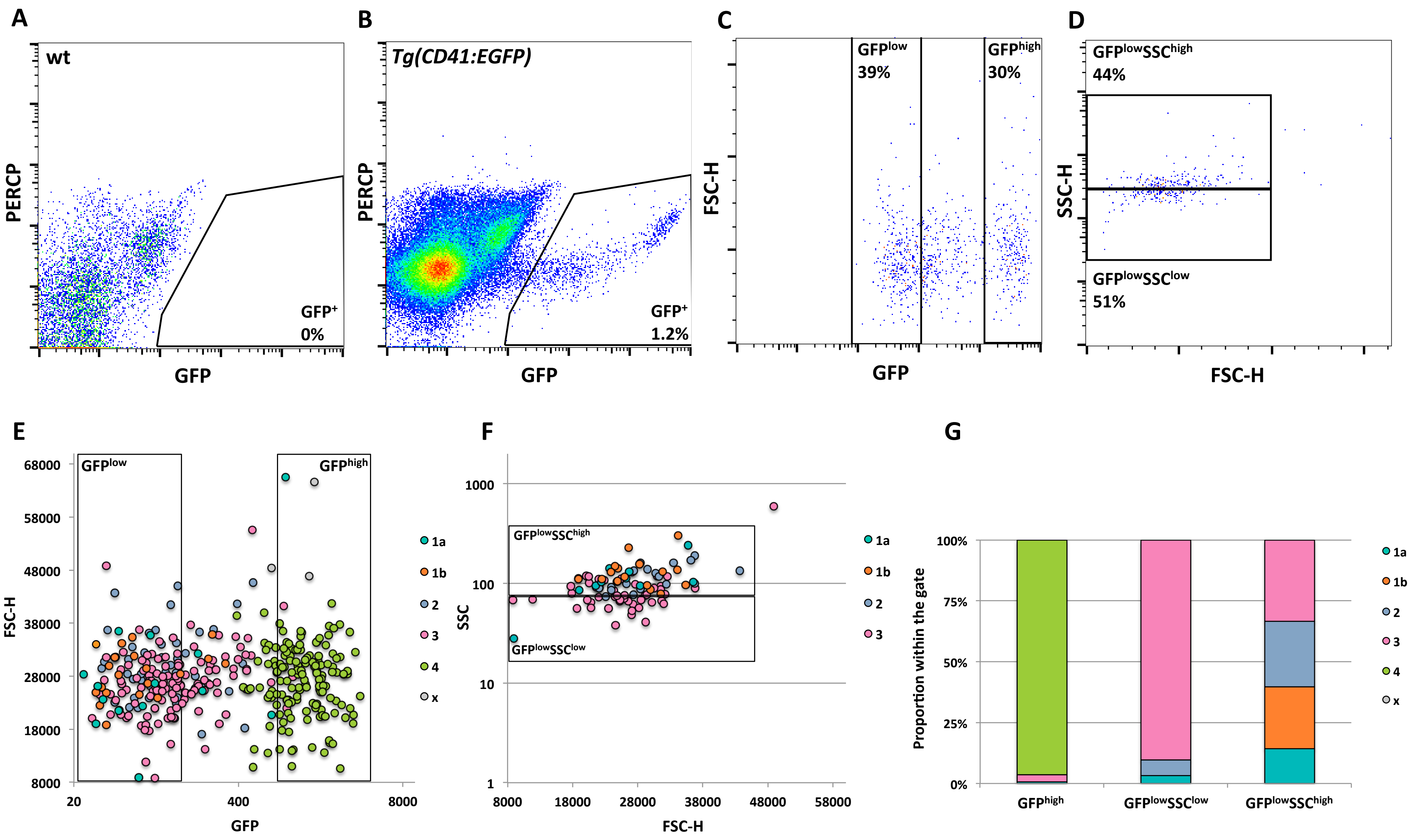
A
wt
PERCP
GFP
GFP+
0%
B
Tg(CD41:EGFP)
PERCP
GFP
GFP+
1.2%
C
FSC-H
GFP
GFPlow
39%
GFPhigh
30%
D
SSC-H
FSC-H
GFPlowSSChigh
44%
GFPlowSSClow
51%
E
FSC-H
GFP
GFPlow
GFPhigh
68000
58000
48000
38000
28000
18000
8000
20
400
8000
1a
1b
2
3
4
x
F
SSC
FSC-H
GFPlowSSChigh
GFPlowSSClow
1000
100
10
1
8000
18000
28000
38000
48000
58000
1a
1b
2
3
G
Proportion within the gate
100%
75%
50%
25%
0%
GFPhigh
GFPlowSSClow
GFPlowSSChigh
1a
1b
2
3
4
x

Figure_S4

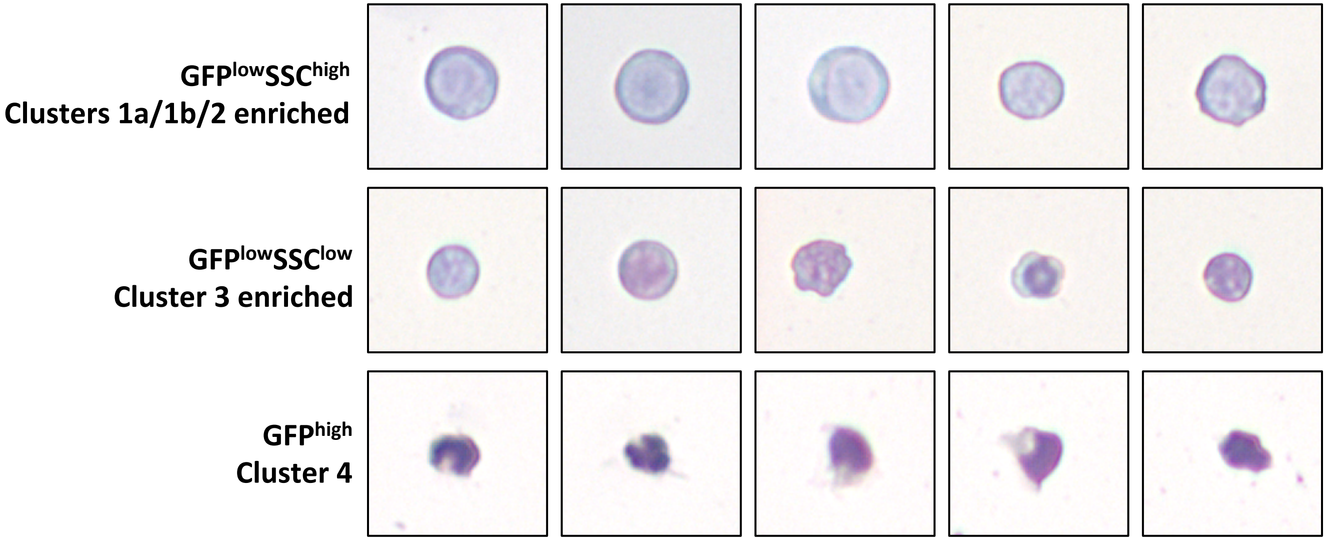
GFP^low SSC^high
Clusters 1a/1b/2 enriched
GFP^low SSC^low
Cluster 3 enriched
GFP^high
Cluster 4

Figure_S5

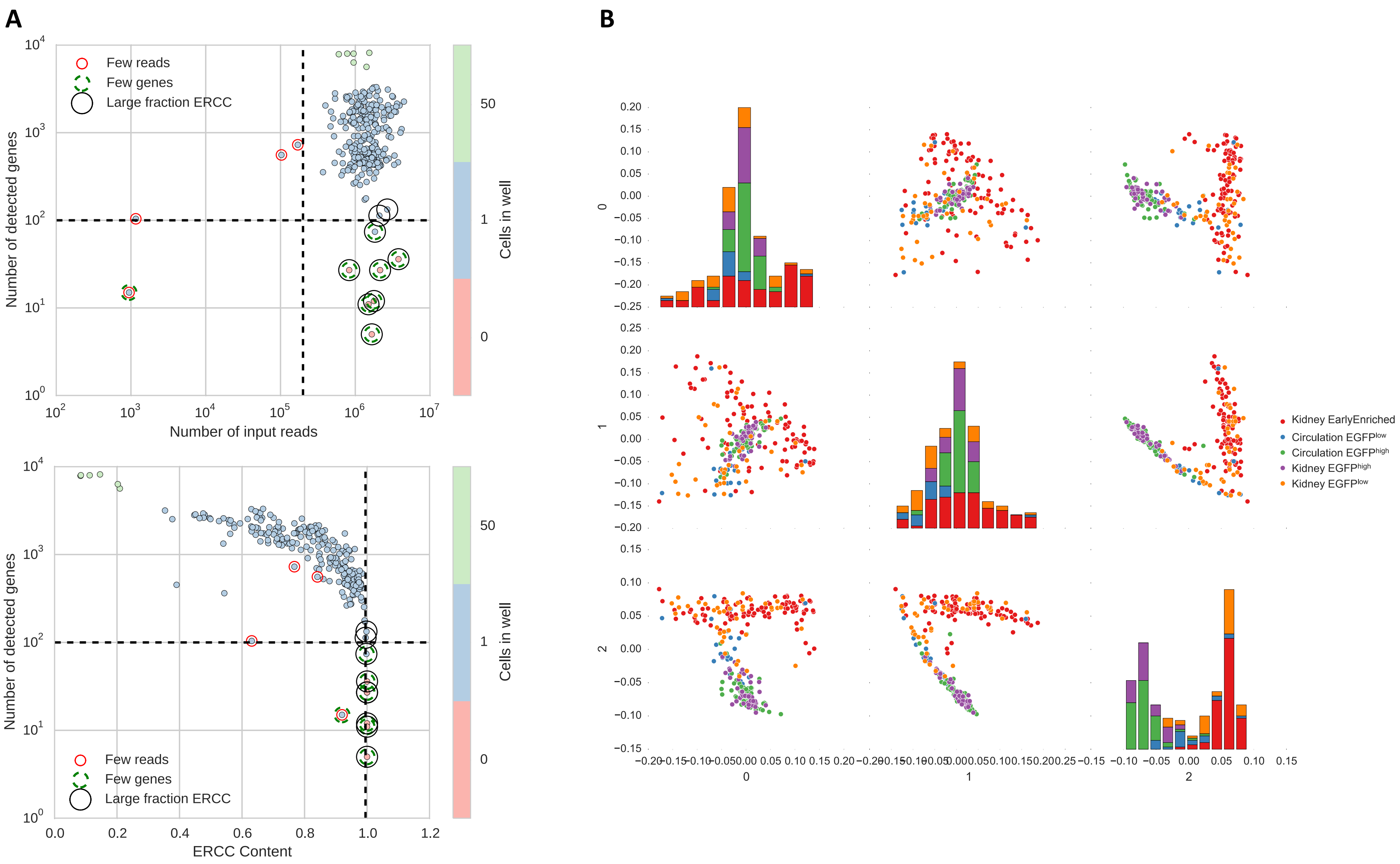

A
Few reads
Few genes
Large fraction ERCC
Number of detected genes
Number of input reads
Cells in well
50
1
0
ERCC Content
B
Kidney EarlyEnriched
Circulation EGFPlow
Circulation EGFPhigh
Kidney EGFPhigh
Kidney EGFPlow

Figure_S6

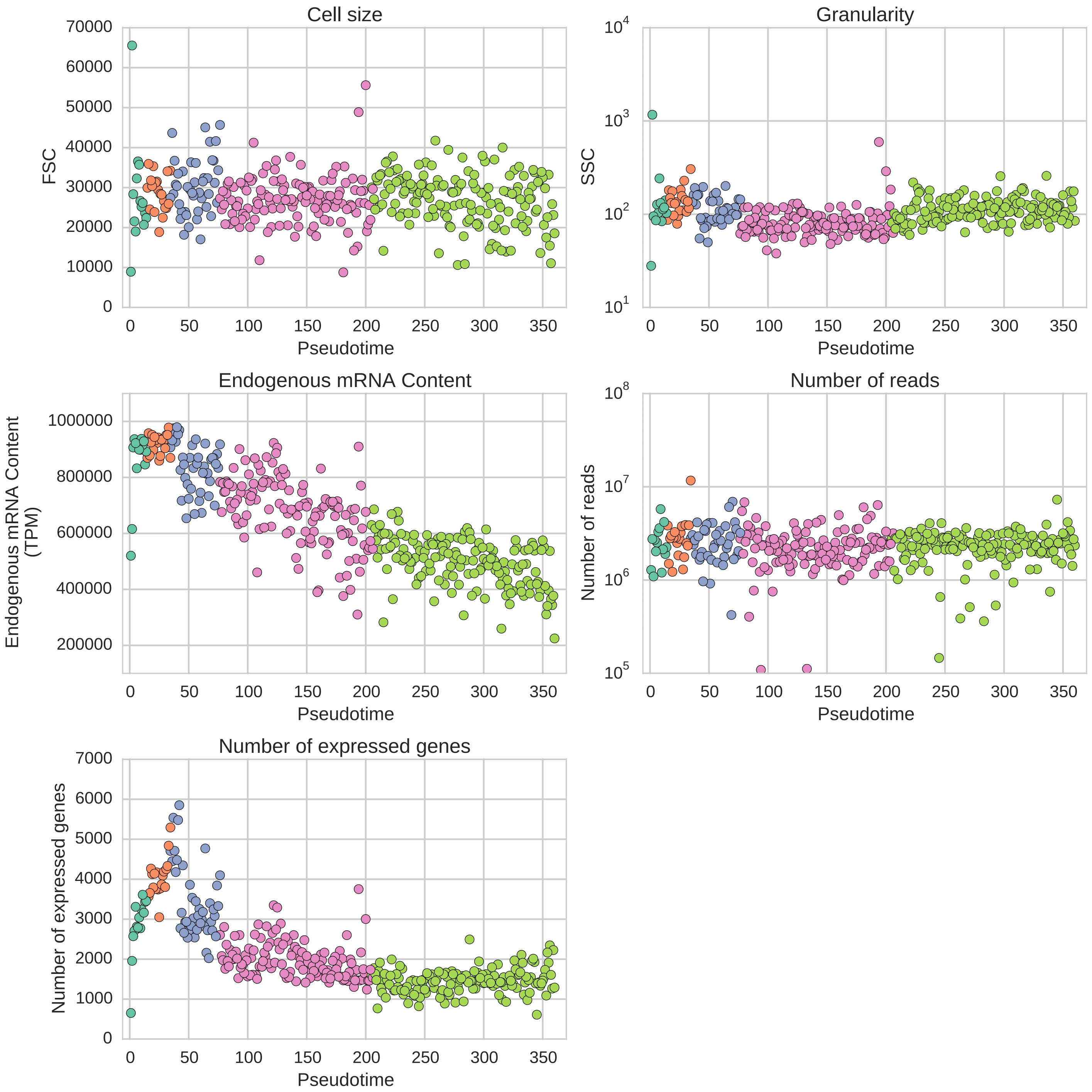

Cell size
FSC
Pseudotime
Granularity
SSC
Pseudotime
Endogenous mRNA Content
Endogenous mRNA Content (TPM)
Pseudotime
Number of reads
Number of reads
Pseudotime
Number of expressed genes
Number of expressed genes
Pseudotime

Figure_S7
