# Supplementary material for: Single-Cell RNA-Sequencing Reveals a Continuous Spectrum of Differentiation in Hematopoietic Cells
Source: Cell Rep. 2016 Jan 21;14(4):966–77. doi: 10.1016/j.celrep.2015.12.082 (PMC4742565; doi:10.1016/j.celrep.2015.12.082)
Supplement: Document S2. Article plus Supplemental Information [file mmc7.pdf]

Resource

Cell Reports

Single-Cell RNA-Sequencing Reveals a Continuous Spectrum of Differentiation in Hematopoietic Cells

Graphical Abstract

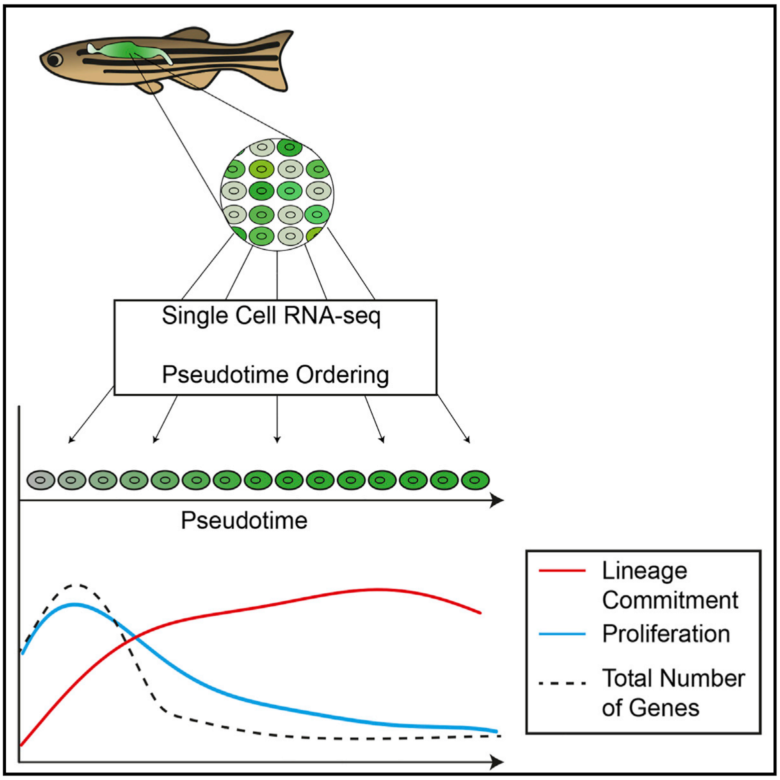

Authors

Iain C. Macaulay, Valentine Svensson, Charlotte Labalette, ..., Thierry Voet, Sarah A. Teichmann, Ana Cvejic

Correspondence

as889@cam.ac.uk

In Brief

Computational reconstruction of the thrombocyte's developmental chronology from scRNA-seq data reveals the continuous nature of the differentiation process. Macaulay et al. show that a highly coordinated transcriptional program characterizes the progression of cells along this continuum. Within this program, there is substantial heterogeneity in the expression of key lineage regulators.

Highlights

- Single-cell RNA-sequencing reveals the continuous nature of thrombocyte development
- Coordinated transcriptional programs govern progression of differentiation
- Number of genes expressed and mRNA content per cell decrease during differentiation
- Zebrafish thrombocytes remain transcriptionally active in circulation

Accession Numbers

E-MTAB-3947

Macaulay et al., 2016, Cell Reports *14*, 966–977
February 2, 2016 ©2016 The Authors
http://dx.doi.org/10.1016/j.celrep.2015.12.082

OPEN ACCESS
CellPress

Cell Reports
Resource

Single-Cell RNA-Sequencing Reveals a Continuous Spectrum of Differentiation in Hematopoietic Cells

Iain C. Macaulay,[1,7] Valentine Svensson,[2,3,7] Charlotte Labalette,[2,4,7] Lauren Ferreira,[2,4] Fiona Hamey,[5] Thierry Voet,[1,6] Sarah A. Teichmann,[2,3,8] and Ana Cvejic[2,4,5,8,*]
[1]Sanger Institute–EBI Single-Cell Genomics Centre, Wellcome Trust Sanger Institute, Wellcome Trust Genome Campus, Hinxton, Cambridge CB10 1HH, UK
[2]Wellcome Trust Sanger Institute, Wellcome Trust Genome Campus, Hinxton, Cambridge CB10 1HH, UK
[3]European Molecular Biology Laboratory, European Bioinformatics Institute (EMBL-EBI), Wellcome Trust Genome Campus, Hinxton, Cambridge CB10 1SD, UK
[4]Department of Haematology, University of Cambridge, Cambridge CB2 0PT, UK
[5]Wellcome Trust – Medical Research Council Cambridge Stem Cell Institute, Cambridge CB2 1QR, UK
[6]Department of Human Genetics, University of Leuven, Leuven 3000, Belgium
[7]Co-first author
[8]Co-senior author
*Correspondence: as889@cam.ac.uk
http://dx.doi.org/10.1016/j.celrep.2015.12.082
This is an open access article under the CC BY license (http://creativecommons.org/licenses/by/4.0/).

SUMMARY

The transcriptional programs that govern hematopoiesis have been investigated primarily by population-level analysis of hematopoietic stem and progenitor cells, which cannot reveal the continuous nature of the differentiation process. Here we applied single-cell RNA-sequencing to a population of hematopoietic cells in zebrafish as they undergo thrombocyte lineage commitment. By reconstructing their developmental chronology computationally, we were able to place each cell along a continuum from stem cell to mature cell, refining the traditional lineage tree. The progression of cells along this continuum is characterized by a highly coordinated transcriptional program, displaying simultaneous suppression of genes involved in cell proliferation and ribosomal biogenesis as the expression of lineage specific genes increases. Within this program, there is substantial heterogeneity in the expression of the key lineage regulators. Overall, the total number of genes expressed, as well as the total mRNA content of the cell, decreases as the cells undergo lineage commitment.

INTRODUCTION

Hematopoietic stem cells (HSCs) have the ability to self-renew and produce cells that give rise to all different blood cell types (Orkin and Zon, 2008). Our understanding of the functional properties of these various hematopoietic cell types has been advanced mainly by population level analysis. Current methods of purifying hematopoietic cells to relative homogeneity are based on the expression of specific combinations of cell surface markers. However, a homogeneous population of cells, as determined by a well-defined set of cell surface markers, may include many functionally distinct populations. This was nicely illustrated in studies showing that within the HSC compartment, individual HSCs may have different reconstitution patterns (e.g., balanced production of myeloid and lymphoid cells or deficiency in lymphoid potential) (Muller-Sieburg et al., 2012). More recently, it was demonstrated that common myeloid progenitors (CMP) are a mixed population of cells with distinct lineage potentials (Notta et al., 2015). The lack of CMPs as a separate cell entity with broad myeloid potential brings into question the traditional model of hematopoietic lineage development and further underscores the importance of revising the current view of lineage development in hematopoiesis. Therefore, there is a need to address the exact composition of the stem and progenitor populations in vivo, as well as the relationships between them. Single cell transcriptome analysis might provide answers to these outstanding questions (Cvejic, 2015).

Among vertebrate models, the zebrafish provides a unique combination of advantages for the study of blood development at the single cell level. Zebrafish blood contains cells of all hematopoietic lineages and orthologs of most transcription factors involved in mammalian hematopoiesis (Hsia and Zon, 2005; Song et al., 2004). Importantly, transcriptional mechanisms and signaling pathways in hematopoiesis are well conserved between zebrafish and mammals, making them a clinically relevant model system (Jagannathan-Bogdan and Zon, 2013).

Over the past few years, a number of transgenic zebrafish lines were generated in which hematopoietic cell specific promoters drive expression of fluorescent molecules (Carradice and Lieschke, 2008). These reporter lines provide a valuable resource of labeled cells ranging from HSCs to a wide range of mature blood cell types. As in mammals, adult hematopoiesis in zebrafish is both continuous and asynchronous. Thus, a single sample of kidney marrow (the analogous tissue to mammalian bone marrow) contains the full spectrum of hematopoietic cell types at various stages of differentiation at any one time. As this is the single site of hematopoiesis in zebrafish, and is easily

OPEN ACCESS
CellPress

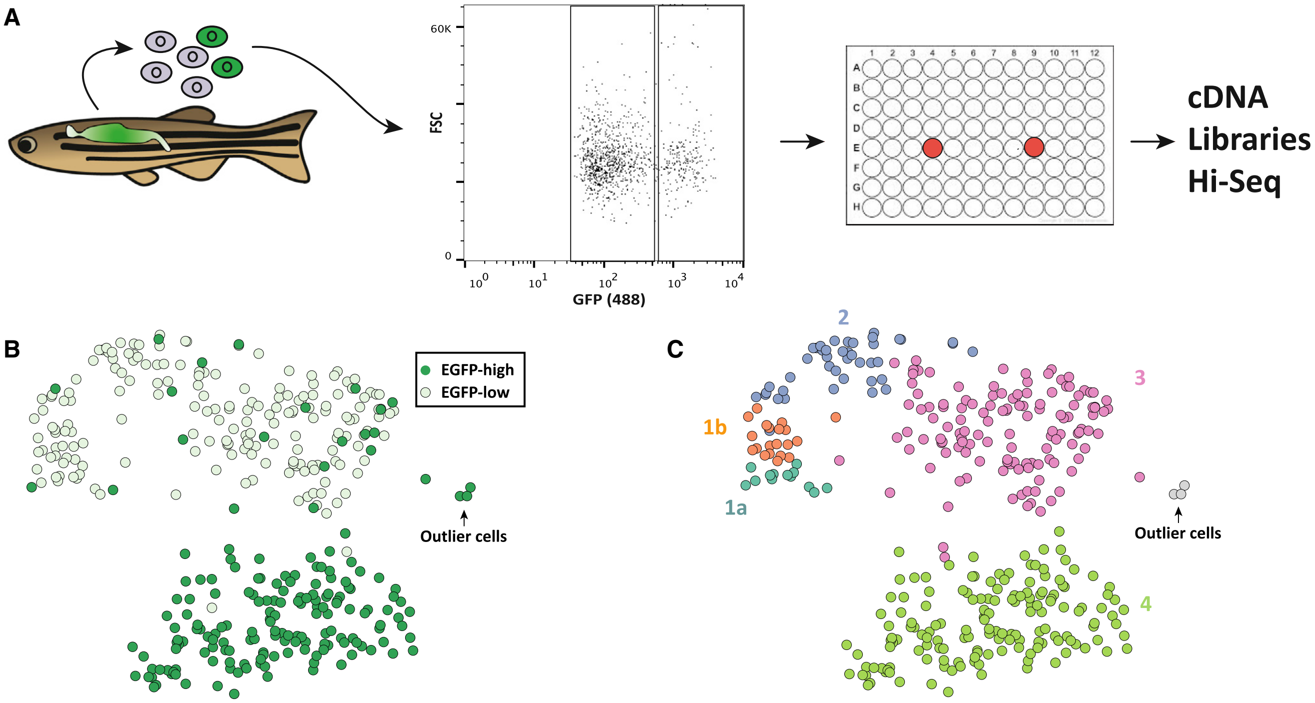

Figure 1. *cd41* Cells Transition through Five Transcriptional States during Thrombocyte Differentiation in Zebrafish

(A) A single kidney, from a heterozygote *Tg(cd41:EGFP)* reporter fish, was dissected and carefully passed through a strainer. Using flow cytometry, EGFP^{low} and EGFP^{high} cells were identified and 188 cells from each population were index sorted. Two wells (in red) per plate were left without cells. RNA from each cell was isolated and used to construct a single mRNA-seq library per cell, which was then sequenced using Hi-seq.

(B) t-SNE plot of the RNA-seq data from 363 EGFP^{low} and EGFP^{high} cells.

(C) The same t-SNE plot (as shown in B) but with points colored based on the cluster the cells belong to. Clusters are labeled as 1a, 1b, 2, 3, 4, and outlier cells. See also Figures S1, S2, and S3.

accessible, the cells are minimally perturbed when sorted ex vivo, making this an ideal system to study basic principles of regulation of differentiation, both at the molecular and cellular levels.

Here we used high-throughput single-cell RNA sequencing combined with fluorescence-activated cell sorting index sorting analysis of adult zebrafish marrow-derived hematopoietic cells. We ordered cells by their progression through differentiation based on gene expression profiles using no prior knowledge of which cell population they belong to, as defined by surface markers. Our analysis revealed the continuous nature of thrombocyte development and the coordinated transcriptional programs that govern differentiation progression. Interestingly, thrombocytes in zebrafish remain transcriptionally active even after leaving the kidney marrow and entering the circulation.

RESULTS

Profiling Individual Hematopoietic Cells Ex Vivo

Here, we used single-cell RNA-sequencing (RNA-seq) of zebrafish kidney cells to resolve the cellular hierarchy of lineage development in the myeloid branch of hematopoiesis. To focus on this lineage, we used expression of CD41 as a marker of HSCs and the megakaryocyte equivalent in fish ("thrombocytes"). CD41 in human is highly regulated during hematopoietic development (Debili et al., 2001; Robin et al., 2011), and in zebrafish, the *Tg(cd41:EGFP)* reporter line labels two distinct populations of cells that express the cd41-EGFP transgene. The weakly fluorescent (EGFP^{low}) subset marks HSCs and progenitor cells (Ma et al., 2011), and the brightly fluorescent (EGFP^{high}) subset includes mature and differentiated thrombocytes (Ma et al., 2011).

Using flow cytometry, we identified EGFP^{low} and EGFP^{high} cells and sorted 188 cells from each population from a single kidney from a *Tg(cd41:EGFP)* reporter fish (Figure 1A; Figures S1A–S1I). Each EGFP^{+} cell was collected in a single well of a 96-well plate, and for each cell, its size (FSC), granularity (SSC), and EGFP fluorescence level were recorded. Single-cell mRNA-seq libraries were constructed and sequenced to a depth of around 2.5 million reads per library. Of 376 cells, 13 cells failed our quality control (QC) and were removed from further analysis (Experimental Procedures; Figures S2A and S2B). For the remaining 363 cells, we accurately quantified between 1,000 and 6,000 genes per cell.

Ordering Hematopoietic Cells from a Single Kidney across Lineage Development

To identify groups of cells and order them in terms of their developmental progression, we used a multi-step approach. First, we used independent component analysis (ICA) to identify distinct factors that describe the variability of EGFP cells. ICA revealed

four latent factors (hidden variables) that explain (1) a progression among EGFP^{low} cells ("within_small_component"), (2) a switch from EGFP^{low} cells toward EGFP^{high} cells ("difference_component"), and (3) progression among the EGFP^{high} cells ("within_large_component"). Finally, the fourth factor identified three outlier cells ("outlier_component") (Figure S3A).

To facilitate data depiction, we used non-linear dimensionality reduction (t-distributed stochastic neighbor embedding [t-SNE]; Van der Maaten and Hinton, 2008) to represent the four latent factors in two dimensions (Figure 1B). ICA revealed a clear distinction between EGFP^{low} and EGFP^{high} cells, implying sharp divergence at the transcriptional level (Figure S3A; Figure 1B).

In addition, EGFP^{low} cells are a more heterogeneous group compared to EGFP^{high} cells. To explore this further, we used hierarchical clustering to partition EGFP cells based on their independent components (Figure S3B). Interestingly, whereas EGFP^{low} cells were split into four distinct clusters (here named 1a, 1b, 2, and 3), EGFP^{high} cells were all grouped into a single cluster (here named 4), confirming the substantial heterogeneity of the EGFP^{low} population of cells (Figure 1C).

Differentiation of hematopoietic cells involves the acquisition of specific phenotypes that depend on the repression of genes characteristic of a multipotent cell state and expression of lineage-restricted genes (Seita and Weissman, 2010). Thus, the whole process can be conceptualized as a temporal ordering of a highly coordinated transcriptional program through which each cell progresses. To examine the transcriptional transitions undergone by cd41-EGFP cells during differentiation, we ordered cells based on the cluster they belonged to, the latent factor that explains the variability of the cells within the cluster, and the level of EGFP fluorescence (details provided in the Experimental Procedures). Our model assumes gradual changes in gene expression during developmental progression of thrombocytes along a one-dimensional (i.e., non-branching) path. (We could not detect any apparent branch point in the data.) This ranking of cells through the entire process was treated as "pseudotime."

To ensure our pseudotime ordering was stable, we also ordered the cells using an alternative method, a Bayesian Gaussian process latent variable model (Titsias and Lawrence, 2010; see Experimental Procedures). Comparing the paths these orderings take when regressed into the t-SNE depiction, one can appreciate the similarity between them (Figure 2A). The two pseudotime orderings agreed very strongly (Spearman correlation 0.97; Figure 2B), giving us confidence in our method.

When presented in pseudotime, the expression of endogenous *cd41* (also known as *itga2b*) and *EGFP*, as well as EGFP fluorescence, recorded during sorting, were highly correlated and showed an expected increase through pseudotime (Spearman rho 0.85, 0.80, and 0.82, respectively) (Figure 2C). This supports our pseudotime ordering of the cells from the HSC to the differentiated thrombocyte extracted from a single kidney.

Inferring Cell States in the Myeloid Lineage

To define the identity of cell types within the five clusters, we evaluated the expression of orthologs of transcription factors and other genes known to be relevant in mammalian hematopoiesis, including the expression of early (cd61, also known as *itgb3a/b*) and late (cd42b, also known as *gp1bb*) markers of megakaryocyte differentiation (Figure 3). The panel of genes analyzed was representative of HSCs (*Tal1*, *Lmo2*, *Lyl1*, *Gata2*, *Runx1*, *Meis1*, *C-myb*, and *Erg*; Capron et al., 2006; Greig et al., 2008; Loughran et al., 2008; Orkin and Zon, 2008; Pineault et al., 2002), megakaryocyte/erythroid (*Fli1*, *Gfi1b*, *Gata1*, *Cd61*, *Cd42b*, *Vwf*, and *Selp*; Clay et al., 2001; Orkin and Zon, 2008; Poirault-Chassac et al., 2010; Schick et al., 1993), and myeloid- (*Gfi1*, *Pu.1* also known as *spi1a/b*, and *Cebp1*; Tenen et al., 1997; Zeng et al., 2004) lineage-affiliated genes.

For each gene, we assessed the level of its expression in pseudotime, as well as the fraction of cells that expressed the gene of interest in each of the clusters (Figure 3). For example, *c-myb* was highly expressed in cluster 1a, as well as in clusters 1b, 2, and 3, but was downregulated in cluster 4. This is in line with previous reports that *C-myb* is expressed in immature hematopoietic cells and is downregulated during differentiation (Greig et al., 2008). Cells in cluster 1a had relatively high expression of *lmo2*, *tal1*, and *meis1*. These genes, together with *fli1*, showed a similar distribution of expression across pseudotime, whereas *gata2* was more restricted to cluster 1a. The mammalian HSC genes *runx1* and *erg* were expressed at a relatively low level overall, and in a small fraction of cells within all clusters. Overall, most of the mammalian HSC marker genes examined are expressed in cluster 1a, and to a lesser degree in 1b, 2, and 3.

In contrast, *Gata1* and *Gfi1b* are known to be expressed at high levels in the erythroid and megakaryocyte lineages (Orkin and Zon, 2008; Vassen et al., 2007) but not in HSCs. In our dataset, *gata1a* and *gfi1b* were expressed in all clusters except cluster 1a. Furthermore, expression of both early (*itgb3a/b*) and late (*gp1bb*) markers of megakaryocyte differentiation started very early and peaked late in pseudotime, confirming that more mature thrombocytes are largely confined to cluster 4.

We also assessed the expression of two well-known platelet genes, *vWf* (von Willebrand factor) and *selp* (P-selectin), through pseudotime (Figure 3). Our analysis revealed that, contrary to previous reports (Carrillo et al., 2010), thrombocytes in zebrafish do not express von Willebrand factor and P-selectin. This was confirmed by qPCR analysis of *cd41* EGFP^{high} thrombocytes from zebrafish kidney. We found, however, that *vWf* was expressed in the whole kidney sample and in fli1:GFP positive cells sorted from *Tg(fli:EGFP)* fish, suggesting that the *vWf* expression pattern differs somewhat in zebrafish compared to mammals.

Surprisingly, myeloid lineage-affiliated genes (e.g., *spi1*, *gfi1*, and *cebp1*) were largely absent across all cells (Figure 3). This suggests that there is no common myeloid progenitor population in this dataset, which charts a continuous HSC to thrombocyte pathway. Altogether, our data are consistent with the notion that cells from cluster 1a belong to HSCs that transition directly to erythroid-thrombocyte progenitor cells, possibly circumventing the CMP step. Although this is surprising, there are other reports of direct, unconventional, HSC to megakaryocyte-erythroid progenitor transitions, such as a recent report in mouse (Guo et al., 2013).

Identification of these progenitor and differentiated cell types prompted us to carry out additional analyses of the sets of genes that strongly correlate with the individual cell types. We used a

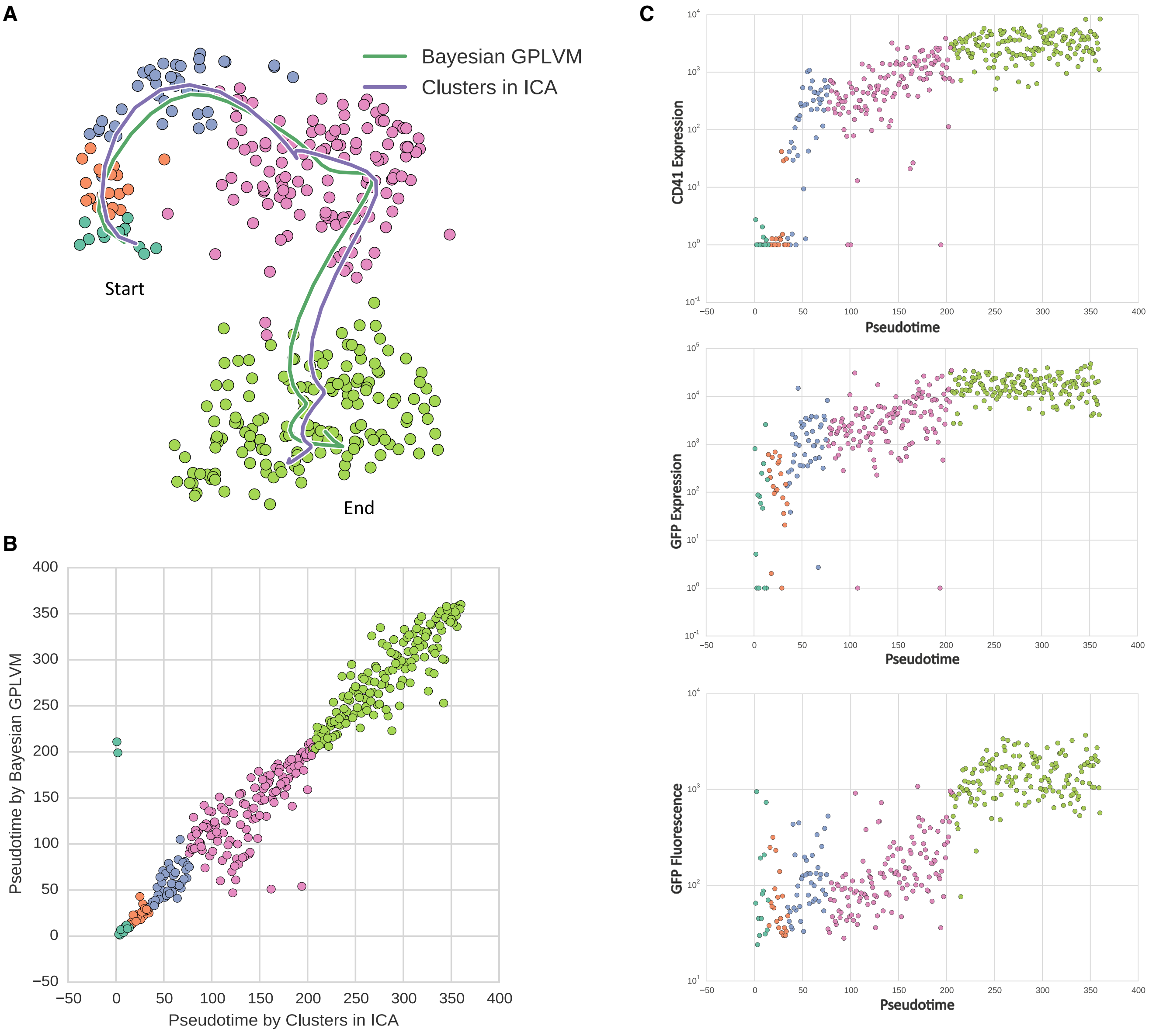

Figure 2. Ordering of Cells through the Developmental Trajectory

(A) We inferred a smooth progression over the developmental lineage, represented as pseudotime, using two different methods. Here we demonstrate the path of both pseudotimes by regressing them into a t-SNE plot of the data. Points are colored based on the cluster the cells belong to.

(B) The pseudotime inferred with two different methods correlate very strongly (Spearman correlation 0.97).

(C) Expression of *cd41* mRNA (top), *GFP* mRNA (middle), and GFP fluorescence (bottom) shown in pseudotime. Each point represents an individual cell; points are colored based on the cluster the cells belong to.

See also Figure S3.

machine learning method, random forest feature importance, to find genes whose expression "marks" distinct clusters of cells. The unique sets of genes expressed in each of the cell types provide an opportunity to reveal novel markers of the identified cell types, and at the same time, provide more insight into their biological function.

Among the numerous newly identified cell-type markers (Table S1), we found several of particular interest (Figure 4A). Ccr9a is a member of the beta chemokine receptor family and is known to be expressed in HSCs (Wright et al., 2002); our data show that *ccr9a* expression is highly correlated with cluster 1a (Figure 4B). Transcription elongation factor A (SII), *tcea3*, was specifically expressed in cluster 1b (Figure 4B). Cells from cluster 1b can also be sorted by combining expression of plasminogen receptor gene (*plgrkt*) and *ascc1* (Figure 4B). Good marker genes for cluster 2 included *e2f8*, which encodes a protein involved in progression through the cell cycle (Deng et al., 2010) and *top2a*, a DNA topoisomerase involved in processes such as chromosome

OPEN ACCESS
CellPress

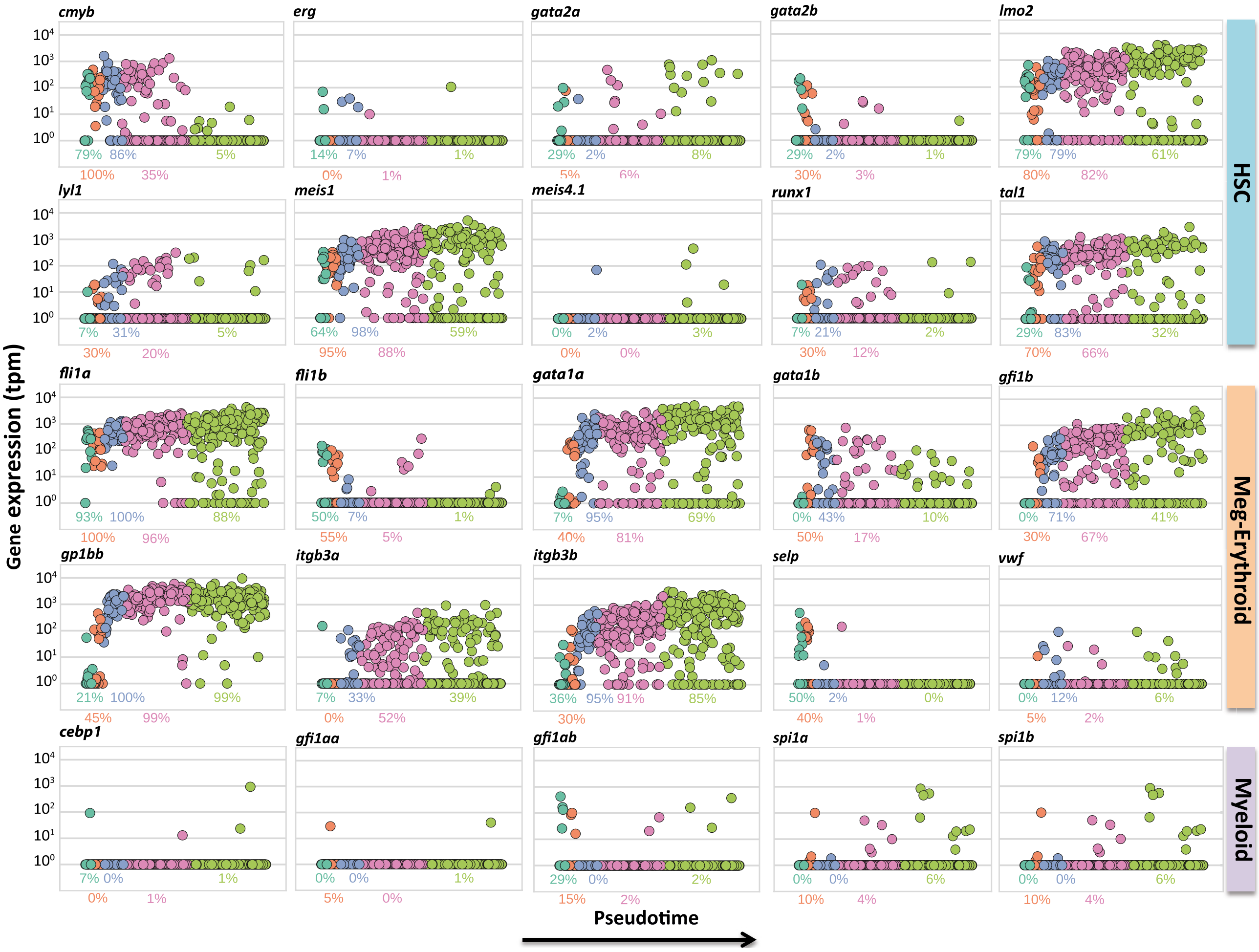

Figure 3. Expression of Key Regulators of Hematopoiesis over Pseudotime

Expression (in TPM) of genes, relevant in hematopoiesis, over pseudotime. Points are colored based on the cluster the cells belong to. For each cluster, we show the proportion of cells within the given cluster expressing the gene at TPM > 1. HSC, hematopoietic stem cells-affiliated genes; Meg-Erythroid, megakaryocyte-erythroid progenitors-affiliated genes; Myeloid, myeloid lineage-affiliated genes.

condensation and chromatid separation (Downes et al., 1994) (Figure 4B). Interestingly, the overrepresented gene ontology (GO) enrichment terms for cluster 2 included cell division and cell cycle (Figure 4A), suggesting that an expansion phase precedes lineage commitment and terminal differentiation of thrombocytes.

To experimentally validate the prediction of greater proliferation in this progenitor population, we sorted cells from clusters 1a/1b/2 versus 3 and 4, by distinguishing these three populations based on EGFP fluorescence, and SSC and FSC (Figures S4A–S4G). We compared the cell cycle distributions of the sorted populations using propidium iodide (PI) staining. The combined cells from clusters 1a/1b/2 had a significantly higher proportion of cells in S and G2/M phase compared to clusters 3 and 4 (Figure 4C), validating our finding that these cells proliferate faster.

These results show that expression of EGFP together with SSC and FSC values could be used to efficiently separate cells from clusters 3 and 4 from the early progenitor populations (1a/1b/2) in the cd41 reporter line (Figures S4 and S5). Additional markers for cluster 3 included combined high expression of *fzd8b* and no expression of *mibp* (Figure 4B). For cluster 4, a high level of cd41 uniquely marks this population.

Finally, we also assessed a unique set of genes expressed by the three outlier cells. GO enrichment analysis of their marker genes yielded only three statistically significant GO terms, all linked with immunity (Figure 4A). One plausible explanation is that these outlier cells represent macrophages that have engulfed or are attached to thrombocytes and hence retained a high level of EGFP fluorescence. Indeed, the outlier cells expressed an array of macrophage/monocyte affiliated genes such as *mpeg* (macrophage expressed gene 1), *csf1r* (colony-stimulating factor 1 receptor), *csf3r* (colony-stimulating factor 3 receptor) etc. Furthermore, compared to all other cells, the outlier cells had remarkably high FSC and SSC values, characteristic of macrophages (Figure 4D).

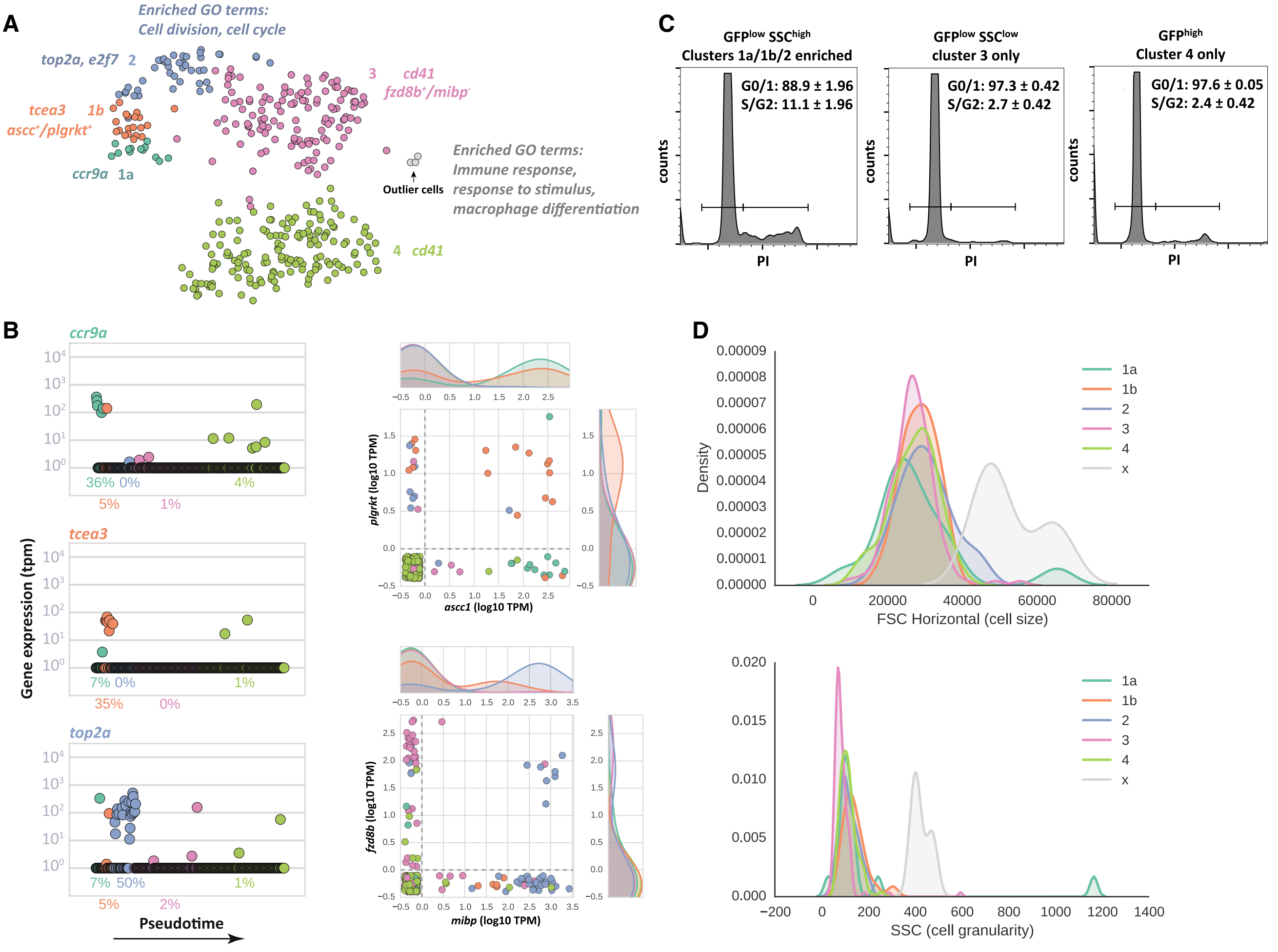

Figure 4. Identification of New Cell-type Markers

(A) t-SNE plot of the RNA-seq data from 363 EGFP cells. Points are colored based on the cluster the cells belong to. Selected genes, whose expression is highly correlated with individual clusters, are shown next to each cluster. Selected gene ontology terms associated with genes that are highly correlated with cluster 2 and the outlier cells are included.

(B) Expression of marker genes over pseudotime (left). Points are colored based on the cluster the cells belong to. For each cluster, we show the proportion of cells expressing the gene at TPM > 1. Expression of pairs of genes is shown on the right. Points are colored based on the cluster the cells belong to. The side diagrams show the proportion of cells within the cluster expressing the gene at the given level of expression.

(C) Cell cycle analysis of three different populations of EGFP cells. The GFP^{low}SSC^{high} cells are enriched for cells from clusters 1a/1b/2, GFP^{low}SSC^{low} and GFP^{high} cells are enriched for cells from clusters 3 and 4, respectively. An average of two experiments is shown as a percentage of cells in G0 and G1 (G0/1) and S and G2 phase (S/G2) ± SEM.

(D) Distribution of FSC (top) and SSC (bottom) values in the different clusters. In particular, one can see that the small population of outliers (cluster x, shaded gray) has higher FSC and SSC values than cells from other clusters.

See also Figures S4 and S5.

Validation of Developmental Progression from the Kidney and Circulation

Importantly, we validated many of our findings in a second set of single cell transcriptomics experiments on kidney cells, as well as circulating cells, from another fish. We sorted an additional 92 cells from cluster 1a/1b/2 (named here EarlyEnriched), 46 EGFP^{low} cells and 46 EFP^{high} cells from the kidney of another *Tg(cd41:EGFP)* fish. We also sorted 24 EGFP^{low} and 68 EGFP^{high} circulating cells from the same fish (Figure S6A). Our analysis confirmed that the pattern of ICA follows the same structure as observed in the previous experiment (Figure S6B). This means that the cell populations and their relative relationships are conserved in this biological replicate. Similarly, the pseudotime ordering of EarlyEnriched, EGFP^{low}, and EGFP^{high} cells in the kidney recapitulated patterns we identified in the initial experiment (Figures 5A–5F).

In addition, we discovered that EGFP^{high} cells in circulation are transcriptionally identical to EGFP^{high} cells in the kidney, with no significant change in the number of expressed genes (Figure 5B), RNA content (Figure 5C), or any gene's expression pattern (likelihood ratio test, corrected for multiple testing with Holm-Sidak). We concluded, therefore, that the thrombocytes exit the kidney

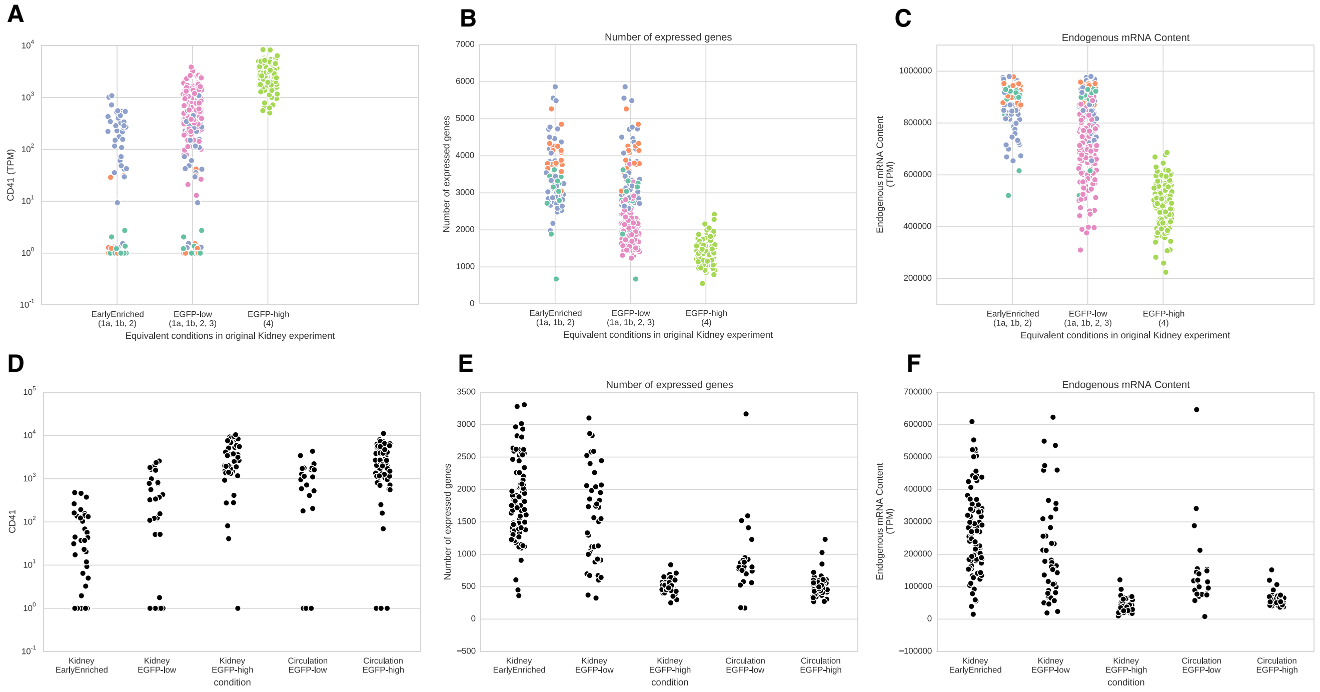

Figure 5. Validation of Identified Early Clusters, and Terminal State of Late Cluster

(A–C) In a second experiment, cells only belonging to the early clusters were sorted. The distributions of (A) *cd41* expression, (B) number of expressed genes, and (C) endogenous mRNA content of cells, are as expected in the populations of cells sorted from kidney.

(D) mRNA expression of *cd41* in the sorted populations of cells. We see the expected increase from Kidney EarlyEnriched through Kidney EGFP^{low} to finally Kidney EGFP^{high}. Expression of *cd41* did not change between Kidney EGFP^{high} and EGFP^{high} cells in circulation (likelihood ratio test, p = 1 after correcting for multiple testing.)

(E and F) When developing from EarlyEnriched through EGFP^{low} to EGFP^{high}, the cells express fewer genes and contain less mRNA, confirming the pseudotime ordering we observed in the initial experiment. There was no change in the number of expressed genes and RNA content between kidney- and circulation-derived EGFP^{high} cells.

See also Figures S6 and S7.

in a fully mature state and are maintained in a transcriptionally active state in circulation.

In both datasets, the total number of genes and total mRNA content expressed per cell were correlated with its differentiation state (Figure S7). This was not due to a difference in the sequencing depth or cell size (Figure S7). Instead, it represents a biological difference between cells during development. This supports the idea that more differentiated, post-mitotic cells (clusters 3 and 4) have a specialized transcriptional program with expression of a small, focused set of genes (Figure S7).

Transcriptional Modules Related to Growth and Proliferation in the Thrombocyte Developmental Gene Expression Program

To find genes with similar trends in expression across pseudotime, we used a mixtures of hierarchical Gaussian processes model to cluster the pseudotime series (Hensman et al., 2015). We identified 130 genes that are dynamically expressed through pseudotime. Clustering of these genes revealed three distinct patterns of their progression during differentiation (Figure 6A; Table S2). Genes upregulated early in pseudotime and then downregulated later (group I) were significantly enriched with the GO term "nucleic acid binding" and "chromosome maintenance" (Figures 6B and 6C; Table S2), possibly reflecting the increased proliferation of cells earlier in pseudotime. Genes gradually downregulated through pseudotime (group II) were highly enriched with the GO terms "eukaryotic translation elongation," "ribosomes" etc. (Figures 6B and 6C; Table S2). Expression of these genes was highly correlated with the general trend of decreased RNA content over pseudotime (Spearman rho = 0.85), therefore suggesting a regulatory loop between the total RNA content in the cell and expression of genes that encode proteins relevant for ribosome synthesis. Finally, genes upregulated early and then maintained at a high level (group III) were highly enriched with the GO terms "ECM-receptor interaction," "platelet aggregation," and "hemostasis," pointing to the genes important for thrombocyte function (Figures 6B and 6C; Table S2). Taken together, our analysis suggests that differentiation of thrombocytes is governed by coordinated transcriptional programs that limit the proliferation of cells and their translational capacity while simultaneously promoting genes relevant for thrombocyte function.

Single Cell Gene Expression Patterns of Whole-Genome Duplicated Genes

Gene duplication is a common event in eukaryotic genomes (Meyer and Schartl, 1999) and due to the teleost-specific

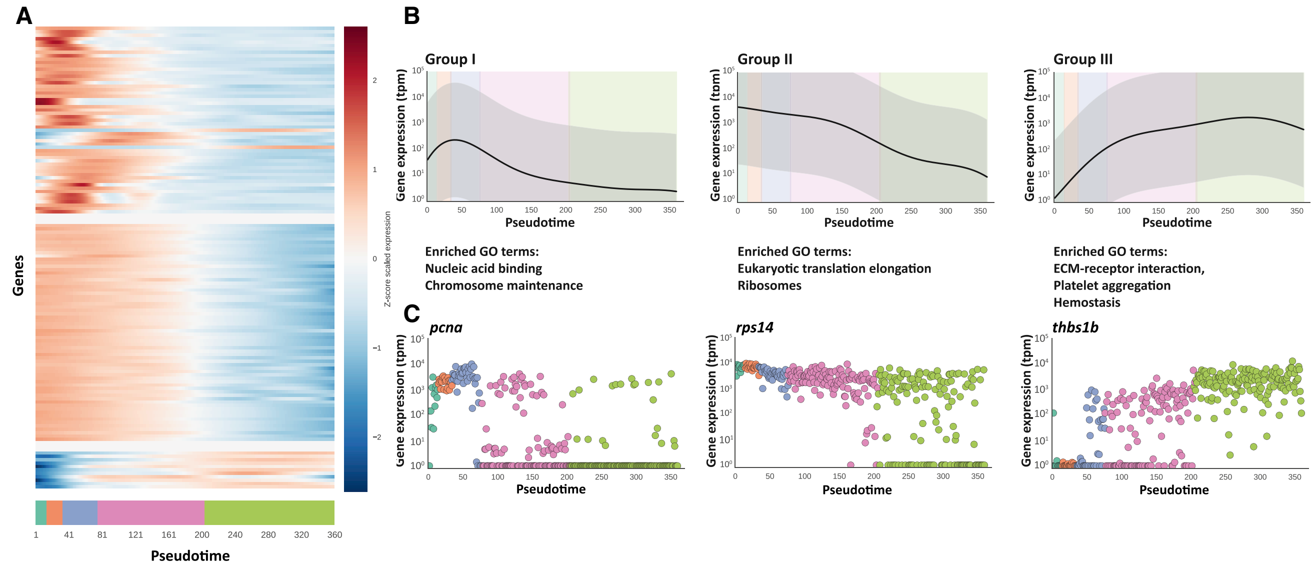

Figure 6. Identification of Genes that Are Dynamically Regulated over Pseudotime

(A) Pseudotime expression patterns of genes (rows) that significantly vary over pseudotime progression (x axis). Every row is the *Z* score scaled Gaussian process representing the expression pattern.

(B) The gene expression pattern for the underlying function explaining the expression pattern in each group is shown as a black line (95% confidence interval in the gray area). Below, selected gene ontology terms associated with the genes in each group are shown.

(C) Expression (in TPM) of an example gene from each group through pseudotime. Points are colored based on the cluster the cells belong to.

genome duplication around 26% (i.e., 3,440) (Howe et al., 2013) of zebrafish genes are duplicated. Gene duplicates that originate from genome duplication are called ohnologs. To assess the use of duplicated genes during thrombopoiesis in zebrafish, we examined the expression of ohnologs in each of the 363 *cd41*:EGFP cells. Of ~8,000 ohnolog categories (Howe et al., 2013), we looked at 3,034 ohnolog categories that have only been duplicated once (ohnolog gene pairs). Of these 3,034 ohnologs (Howe et al., 2013), 2,107 were not expressed in our dataset. However the remaining 927 pairs can be divided into the following three major groups: (1) expression of ohnologs is mutually exclusive in individual cells (n = 177) (Figures 7A and 7B; Table S3). In this group, the expression of any one ohnolog appeared to be an independent event with an equal probability of happening. This suggests selective activation or silencing of these ohnologs in individual cells; (2) only one of the ohnologs is expressed in all cells (n = 430), Figures 7A and 7B; Table S3), and (3) both ohnologs are equally expressed in all cells (n = 218), (Figures 7A and 7B; Table S3). No patterns of ohnolog use over pseudotime were observed.

DISCUSSION

Here we show the power of single cell transcriptome analysis to decipher the kinetics of hematopoietic lineage development. We ordered cd41 cells by their progression through differentiation based on gene expression profiles. Our analysis illustrates the continual nature of this process, where cells progressively transit through five transcriptional states that result in the generation of mature thrombocytes.

Interestingly, myeloid lineage-affiliated genes were largely absent across all cells, suggesting direct HSC to thrombocyte-erythroid progenitor transition. The model of hematopoiesis generated recently, using single cells from over ten hematopoietic populations in mouse, implies that the megakaryocyte-erythroid lineage is closely linked to long-term repopulating HSCs and separates early from the lympho-myeloid lineage (Guo et al., 2013). The identification of platelet-primed stem cells within vWf-expressing long-term HSCs further confirmed that commitment to the megakaryocyte lineage starts in the most primitive stem cell compartment (Sanjuan-Pla et al., 2013). Although in our dataset vWf was not expressed in any of the identified cell populations, the low expression of some of the thrombocyte lineage-affiliated genes in cluster 1a suggests that using our sorting strategy we are possibly capturing thrombocyte-primed stem cells. Therefore, HSCs in cluster 1a may represent a biased subpopulation within the wider pool of hematopoietic stem/progenitor cells present in the zebrafish kidney. Nevertheless, the gradual transition of cells during thrombocyte lineage development that we see in our dataset (e.g., gradual changes in the total number of genes as well as the total mRNA content) suggest that we do capture a continuous spectra of cells and that the common myeloid stage is not an obligatory step during thrombopoiesis.

We also show that although each of the identified transcriptional states was characterized by substantial heterogeneity in the expression of the key lineage regulators, the underlying transcriptional program was highly coordinated. It included the simultaneous increase in the expression of genes important

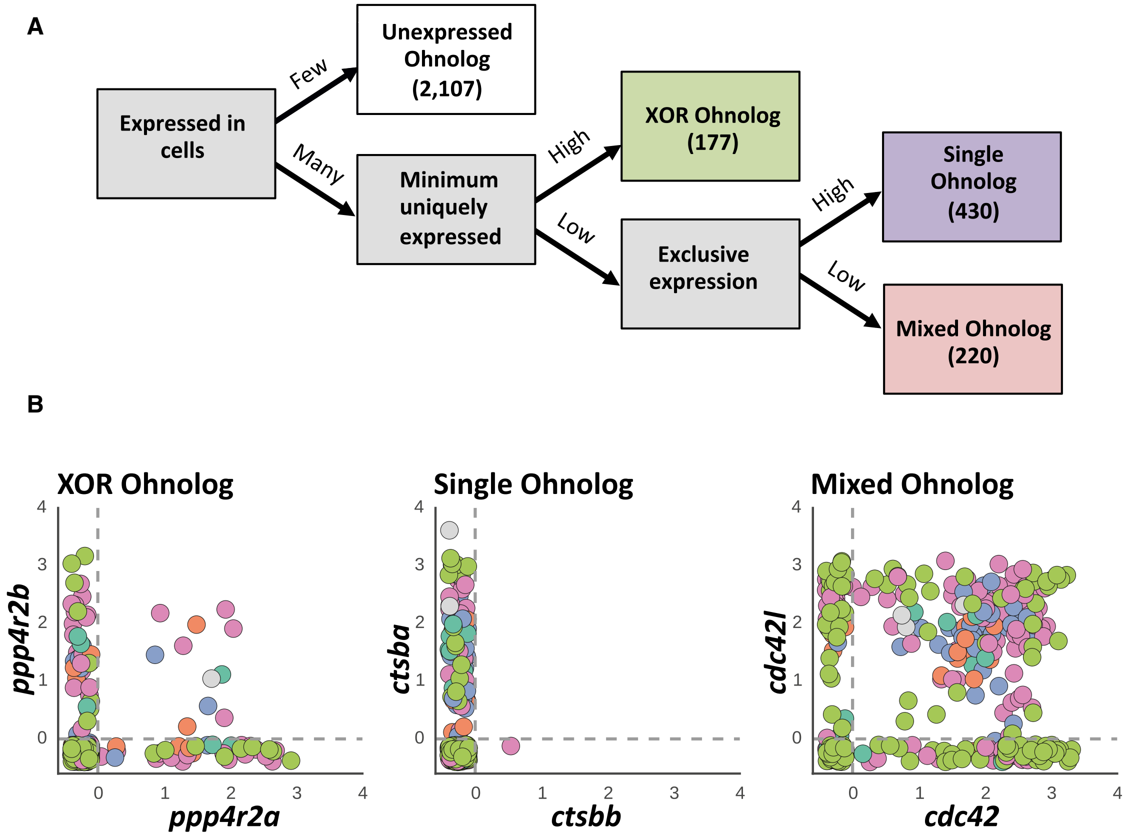

Figure 7. Single Cell Analysis Reveals Three Main Patterns of Usage of Duplicated Genes during Thrombopoiesis in Zebrafish

(A) Ohnolog gene pairs were divided into four classes based on thresholds in a decision tree.

(B) Expression (in TPM) of example ohnologs, randomly selected from each class, in individual cells. Points are colored based on the cluster the cells belong to. XOR ohnolog: both ohnologs are expressed but never in the same cell. Single ohnolog: just one ohnolog is expressed. Mixed ohnologs: both ohnologs are expressed in individual cells.

for thrombocyte function and suppression of genes relevant in cell proliferation and ribosomal biogenesis. Interestingly, although the maturation of thrombocytes was completed in the kidney, they maintained a transcriptionally active state in circulation. We did not, however, detect any qualitative or quantitative difference in the gene expression between circulating and kidney-based EGFP^{high} thrombocytes. Surprisingly, unlike mammalian platelets, which have abundant expression of vWf, thrombocytes in zebrafish do not express vWf. Instead, our analysis suggests that other cells within the kidney marrow, such as endothelial cells (*fli1:GFP* positive cells), express vWf in zebrafish. Finally, we assessed use of duplicated genes during thrombopoiesis in zebrafish and identified patterns of their expression that would not be possible using a bulk transcriptomics approach.

We used single-cell RNA-seq of zebrafish kidney cells to resolve the cellular hierarchy of lineage development in the myeloid branch of hematopoiesis and propose a refined model of developmental progression of hematopoietic cells.

Our study addresses some of the basic questions of regulation of differentiation, both at the molecular and cellular levels. In this study, we focused on zebrafish thrombocyte development; however, a similar approach could be used in other systems and cell types.

EXPERIMENTAL PROCEDURES

Zebrafish Strains and Maintenance

The maintenance of wild-type (Tubingen Long Fin) and transgenic zebrafish Tg(*cd41:GFP*) lines were performed in accordance with EU regulations on laboratory animals, as previously described (Bielczyk-Maczyńska et al., 2014).

Single-Cell Sorting and Whole Transcriptome Amplification

A single kidney from heterozygote *Tg*(*cd41:EGFP*) or wild-type fish was dissected and carefully passed through a strainer using the plunger of a 1 ml syringe. In the follow-up experiment, circulating GFP-positive cells were collected from the dissected heart of the same fish. Cells were collected in cold 1× PBS/5% fetal bovine serum. The kidney of a non-transgenic line was used to set up the gating and exclude autofluorescent cells. Dead cells were excluded based on PI staining. Individual cells were sorted using a Becton Dickinson Influx sorter with 488- and 561-nm lasers (Schulte et al., 2015) and collected in a single well of a 96-well plate containing 2.3 μl of 0.2% Triton X-100 supplemented with 1 U/μl SUPERase In RNase inhibitor (Ambion). At the same time, information about cell size and granularity and the level of the fluorescence were recorded. Whole transcriptome amplification and library preparation was performed using the Smart-seq2 protocol (Picelli et al., 2014,

2013), with ERCC spike-in controls added at the same time as the oligo-dT and dNTP mixture. Twenty-five PCR cycles were performed during the amplification.

Cell Cycle Analysis

GFP-positive cells from *Tg*(*cd41:EGFP*) kidney suspension were sorted using a Mo-Flo XDP (Beckman Coulter) with 488-, 561-, and 640-nm lasers. Cells were centrifuged at 1,200 rpm for 10 min at 4°C, resuspended in 100 μl 1× PBS and fixed by adding 300 μl ethanol. Cells were fixed overnight at 4°C, washed twice in 1× PBS, and re-suspended in 500 μl PI solution (25 μg/ml PI, 0.1% Triton X-100, 0.1% sodium citrate). Cells were incubated for 3 hr with RNase A (Sigma) and analyzed by BD LSR Fortessa (Becton Dickinson). Data were analyzed using FlowJo software.

Cytology

Sorted EGFP-positive cells were concentrated by cytocentrifugation at 350 rpm for 5 min onto SuperFrostPlus slides using a Shandon Cytospin 3 cytocentrifuge. Slides were fixed for 3 min in methanol and stained with May-Grünwald Giemsa (Sigma) as described elsewhere (Stachura et al., 2009). Images were captured as described elsewhere (Bielczyk-Maczyńska et al., 2014).

Verification of RNA-Seq Data with qPCR

GFP-positive cells from *Tg*(*cd41:EGFP*) and *Tg*(*fli1:EGFP*) kidney suspensions were sorted using a Mo-Flo XDP (Beckman Coulter), along with an equal number of viable cells from the whole kidney, into 75 μl RLT buffer (QIAGEN) containing 1% β-mercaptoethanol. mRNA was extracted using Oligo $(dT)_{25}$ Dynabeads (Ambion) and cDNA was prepared using SuperScript VILO (Invitrogen), according to the manufacturers' instructions. qPCR reactions were performed using the 7900HT Real Time system (Life Technologies) with primers for *vWf* (F: CGGCAGCACATACACACATT and R: CGTTCCATCCACAGAGAGGT) and two housekeeping genes (eif1a F: GAGAAGTTCGAGAAGGAAGC and R: CGTAGTATTTGCTGGTCTCG, and b-actin F: CGAGCAGGAGATGGG AACC and R: CAACGGAAACGCTCATTGC). The ΔΔCt method was used for data analysis.

Single-Cell RNA-Seq Data Processing

Reads from RNA-seq were aligned to the zebrafish genome (Zv9.77) combined with sequences for eGFP and ERCC spike-ins as artificial chromosomes, using STAR (version 2.3; (Dobin et al., 2013). The Ensembl Genes annotation track from UCSC was used with the read_distribution.py tool from the RSeQC tool suite (Wang et al., 2012) to generate quality control information. Gene expression was quantified using the Salmon (Patro et al., 2015) reads mode of Sailfish (Patro et al., 2014; parameter -l IU) using Zv9 cDNA sequences from Ensembl version 77 as transcript sequences, together with ERCC spike-in and eGFP sequences as artificial transcripts. Based on comparison with empty control wells, samples with less than 50,000 paired reads and 1,000 expressed genes were considered unfit and were excluded from further analysis (Figure S2).

For the follow-up experiment, expression was quantified the same way. We used a different stock and concentration of ERCC spike-ins, which changed the scales of the QC values. For these samples, we excluded cells with less than 200,000 paired reads and less than 150 expressed genes (Figure S6).

Downstream analysis was performed using Transcripts per million (TPM) values reported by Salmon. The TPM unit is a measure of relative abundance of a gene, which is stable across samples (Li and Dewey, 2011; Wagner et al., 2012). Before analysis expression for endogenous spike-ins were filtered out for each cell, and the TPM for each cell was rescaled to sum to a million. This gives us the interpretation that TPM of a gene will correspond to the concentration of mRNAs from a gene in a given cell.

Unless stated otherwise, for all analyses, we filtered out genes expressed at a level higher than 1 TPM in only less than three cells, which leaves 20,556 genes.

Identifying Processes and Ordering Cells by Hidden Factors

We used ICA (Hyvärinen and Oja, 2000) to identify four latent factors (hidden variables modeling the data), as implemented in scikit-learn (with parameter random_state = 3,984 for the sake of reproducibility). The choice of four components was based on testing between one and ten components, and seeing diminishing returns on the Frobenius norm reconstruction error past four components. One latent factor explains a progression among $EGFP^{low}$ cells; another factor explains a switch from $EGFP^{low}$ cells toward the population of $EGFP^{high}$ cells. A third factor explains progression among $EGFP^{high}$ cells. The fourth factor identifies three outlier cells. We used the fluorescence levels of GFP to flip the orientation of the latent factors so that a higher factor value always corresponded to a higher GFP value. Because these factors are orthogonal, they are statistically independent. In other words, there are three distinct processes happening sequentially. We performed hierarchical Ward clustering (Ward, 1963) of the cells in the four-dimensional ICA space, and assigned the cells to six clusters. (For exact commands, see Notebook 1 in Data S2.) Based on which cluster the cells belonged to, and which factor explains the variability of the cells of that cluster, we ordered cells along this three-stage progression. This ranking of cells through the entire process was treated as pseudotime. (For exact commands, see Notebook 3 in Data S2.)

As an alternative way to estimate a pseudotime, we applied a Bayesian Gaussian process latent variable model with a one-dimensional latent variable (Titsias and Lawrence, 2010). Briefly, the Bayesian GPLVM will infer a nonlinear function from an unobserved latent space to an observed high-dimensional space, using inducing inputs that are variationally inferred, which helps smooth the data and speed up computation. In our case, the latent space is the one-dimensional pseudotime, and the non-linear function will be a mapping from pseudotime to gene expression values. We used the BayesianGPLVM implementation in the GPy package (The GPy authors, n.d.) using a Radial Basis Function (RBF) kernel on the log-transformed TPM values, all other parameters default. Without any information about the EGFP expression, the BayesianGPLVM recovers our original ordering, up to orientation (Spearman correlation 0.97; Figure 2B) (Notebook 7 in Data S2).

To depict the structure of the data in a friendly way, we performed t-distributed stochastic neighbor embedding (t-SNE) (Van der Maaten and Hinton, 2008) of the four latent factors into two dimensions. The goal of the t-SNE algorithm is to attempt to preserve both global and local structures of higher dimensional data in two dimensions. It additionally tries to not crowd areas with too many points, making them hard to see. We set the perplexity parameter to 75 and used a fixed random seed to make sure the t-SNE plot would be reproducible (parameter random_state = 254 in the scikit-learn implementation of t-SNE).

We can depict the inferred pseudotime by regressing it into the two-dimensional tSNE space (Figure 2A) and can see how well the two methods of constructing pseudotime agrees.

Marker Gene Discovery

To discover marker genes for the clusters of cells, we trained a random forest model for each cluster versus the rest of the cells. We used the Gini feature importance scores for each gene to order genes by how well they can distinguish a cluster from the rest of the cells. We used the ExtraTreesClassifier (Geurts et al., 2006) implementation in the Python machine learning package scikit-learn (Pedregosa et al., 2011), with the parameter n_estimators = 100,000. (For the exact commands, see Notebook 2 in Data S2.)

Pseudotime Analysis

We treated the pseudotime progression order of the cells as a time series, and for each gene trained two Gaussian processes (GPs): one with a radial basis function (RBF) kernel (which can model change over time) and one with a constant kernel (which assumes that the expression of a gene does not change over time). After optimizing parameters for both models, we filtered the genes by the ratios of the likelihoods of the models. If the RBF kernel GP has a higher likelihood than the constant kernel GP, we can conclude that the gene in question has expression that is dynamic in time. Once we had identified genes that were pseudotime-dependent, we applied the mixtures of hierarchical Gaussian processes model to identify groupings of genes with similar pseudotime expression patterns (Hensman et al., 2015). All functional enrichment analysis was performed with the gProfiler (Reimand et al., 2011) web service with the standard gene list as background (see Notebook 4 in Data S2 for exact commands).

Classification of Ohnolog Gene Pairs

We obtained the list of duplicated genes arising from the teleost-specific genome duplication event from (Howe et al., 2013). We filtered the list to only retain pairs of genes whose IDs were present in version 77 of Ensembl. For these genes, we binarized the expression to "expressed" or "not expressed" in each cell based on whether the TPM was greater than 1. Using these binary values, for each Ohnolog pair we counted cells expressing either member of the pair, both members of the pair, or none of the members in the pair. Ohnolog pairs in which none of the members were expressed in more than 300 cells and were annotated as "Not expressed." We defined a value "both_min_diff" as the difference between the smallest number of cells expressing only one of the members in a pair, and the number of cells expressing both members of the pair. Ohnolog pairs with a "both_min_diff"-value larger than 15 were annotated as "XOR Ohnologs." To identify Ohnolog pairs in which only one member was used, we looked at the difference between the largest number of cells using one member compared to the largest number of cells using the other member. If this difference was larger than 60 cells, the Ohnolog pair was considered a "Single Ohnolog." The remaining cells were dubbed "Mixed Ohnologs," meaning cells with a mixture of both members of a pair. (See Notebook 5 in Data S2 for exact commands.)

All analysis scripts are provided as IPython notebooks in the supplemental information (Data S1, Sample Information) together with a table of detailed information of each sample (Data S2, Analysis Files).

ACCESSION NUMBERS

The accession number for the data reported in this paper is ArrayExpress: E-MTAB-3947.

SUPPLEMENTAL INFORMATION

Supplemental Information includes seven figures, three tables, and two data files and can be found with this article online at http://dx.doi.org/10.1016/j.celrep.2015.12.082.

AUTHOR CONTRIBUTIONS

I.C.M, C.L., and L.F. performed experiments. V.S. carried out the analysis. I.C.M, C.L., V.S., F.H., S.T., and A.C. contributed to the discussion of the results. T.V. oversaw implementation of the scRNA-seq pipeline. I.C.M, C.L., V.S., and A.C. wrote the manuscript. S.H. edited the manuscript. A.C. conceived the study. All authors approved the final version of the manuscript.

ACKNOWLEDGMENTS

We thank the Sanger-EBI single cell centre and Chiara Cossetti, Michal Maj, and Reiner Schulte at the CIMR flow cytometry Core for their help with index cell sorting. The study was supported by Cancer Research UK grant number C45041/A14953 (to A.C., C.L., and L.F.) and a core support grant from the Wellcome Trust and MRC to the Wellcome Trust – Medical Research Council Cambridge Stem Cell Institute. T.V. acknowledges the Wellcome Trust and KU Leuven (SymBioSys, PFV/10/016). S.T would like to acknowledge the Lister Research Prize from the Lister Institute.

Received: August 10, 2015
Revised: October 30, 2015
Accepted: December 16, 2015
Published: January 21, 2016

REFERENCES

Bielczyk-Maczyńska, E., Serbanovic-Canic, J., Ferreira, L., Soranzo, N., Stemple, D.L., Ouwehand, W.H., and Cvejic, A. (2014). A loss of function screen of identified genome-wide association study Loci reveals new genes controlling hematopoiesis. PLoS Genet. *10*, e1004450.

Capron, C., Lécluse, Y., Kaushik, A.L., Foudi, A., Lacout, C., Sekkai, D., Godin, I., Albagli, O., Poullion, I., Svinartchouk, F., et al. (2006). The SCL relative LYL-1 is required for fetal and adult hematopoietic stem cell function and B-cell differentiation. Blood *107*, 4678–4686.

Carradice, D., and Lieschke, G.J. (2008). Zebrafish in hematology: sushi or science? Blood *111*, 3331–3342.

Carrillo, M., Kim, S., Rajpurohit, S.K., Kulkarni, V., and Jagadeeswaran, P. (2010). Zebrafish von Willebrand factor. Blood Cells Mol. Dis. *45*, 326–333.

Clay, D., Rubinstein, E., Mishal, Z., Anjo, A., Prenant, M., Jasmin, C., Boucheix, C., and Le Bousse-Kerdilès, M.C. (2001). CD9 and megakaryocyte differentiation. Blood *97*, 1982–1989.

Cvejic, A. (2015). Mechanisms of fate decision and lineage commitment during haematopoiesis. Immunol. Cell Biol. http://dx.doi.org/10.1038/icb.2015.96

Debili, N., Robin, C., Schiavon, V., Letestu, R., Pflumio, F., Mitjavila-Garcia, M.T., Coulombel, L., and Vainchenker, W. (2001). Different expression of CD41 on human lymphoid and myeloid progenitors from adults and neonates. Blood *97*, 2023–2030.

Deng, Q., Wang, Q., Zong, W.-Y., Zheng, D.-L., Wen, Y.-X., Wang, K.-S., Teng, X.-M., Zhang, X., Huang, J., and Han, Z.-G. (2010). E2F8 contributes to human hepatocellular carcinoma via regulating cell proliferation. Cancer Res. *70*, 782–791.

Dobin, A., Davis, C.A., Schlesinger, F., Drenkow, J., Zaleski, C., Jha, S., Batut, P., Chaisson, M., and Gingeras, T.R. (2013). STAR: ultrafast universal RNA-seq aligner. Bioinformatics *29*, 15–21.

Downes, C.S., Clarke, D.J., Mullinger, A.M., Giménez-Abián, J.F., Creighton, A.M., and Johnson, R.T. (1994). A topoisomerase II-dependent G2 cycle checkpoint in mammalian cells/. Nature *372*, 467–470.

Geurts, P., Ernst, D., and Wehenkel, L. (2006). Extremely randomized trees. Mach. Learn. *63*, 3–42.

Greig, K.T., Carotta, S., and Nutt, S.L. (2008). Critical roles for c-Myb in hematopoietic progenitor cells. Semin. Immunol. *20*, 247–256.

Guo, G., Luc, S., Marco, E., Lin, T.-W., Peng, C., Kerenyi, M.A., Beyaz, S., Kim, W., Xu, J., Das, P.P., et al. (2013). Mapping cellular hierarchy by single-cell analysis of the cell surface repertoire. Cell Stem Cell *13*, 492–505.

Hensman, J., Rattray, M., and Lawrence, N.D. (2015). Fast Nonparametric Clustering of Structured Time-Series. IEEE Trans. Pattern Anal. Mach. Intell. *37*, 383–393.

Howe, K., Clark, M.D., Torroja, C.F., Torrance, J., Berthelot, C., Muffato, M., Collins, J.E., Humphray, S., McLaren, K., Matthews, L., et al. (2013). The zebrafish reference genome sequence and its relationship to the human genome. Nature *496*, 498–503.

Hsia, N., and Zon, L.I. (2005). Transcriptional regulation of hematopoietic stem cell development in zebrafish. Exp. Hematol. *33*, 1007–1014.

Hyvärinen, A., and Oja, E. (2000). Independent component analysis: algorithms and applications. Neural Netw. *13*, 411–430.

Jagannathan-Bogdan, M., and Zon, L.I. (2013). Hematopoiesis. Development *140*, 2463–2467.

Li, B., and Dewey, C.N. (2011). RSEM: accurate transcript quantification from RNA-seq data with or without a reference genome. BMC Bioinformatics *12*, 323.

Loughran, S.J., Kruse, E.A., Hacking, D.F., de Graaf, C.A., Hyland, C.D., Willson, T.A., Henley, K.J., Ellis, S., Voss, A.K., Metcalf, D., et al. (2008). The transcription factor Erg is essential for definitive hematopoiesis and the function of adult hematopoietic stem cells. Nat. Immunol. *9*, 810–819.

Ma, D., Zhang, J., Lin, H.-F., Italiano, J., and Handin, R.I. (2011). The identification and characterization of zebrafish hematopoietic stem cells. Blood *118*, 289–297.

Meyer, A., and Schartl, M. (1999). Gene and genome duplications in vertebrates: the one-to-four (-to-eight in fish) rule and the evolution of novel gene functions. Curr. Opin. Cell Biol. *11*, 699–704.

Muller-Sieburg, C.E., Sieburg, H.B., Bernitz, J.M., and Cattarossi, G. (2012). Stem cell heterogeneity: implications for aging and regenerative medicine. Blood *119*, 3900–3907.

Notta, F., Zandi, S., Takayama, N., Dobson, S., Gan, O.I., Wilson, G., Kaufmann, K.B., McLeod, J., Laurenti, E., Dunant, C.F., et al. (2015). Distinct routes of lineage development reshape the human blood hierarchy across ontogeny. Science, aab2116.

Orkin, S.H., and Zon, L.I. (2008). Hematopoiesis: an evolving paradigm for stem cell biology. Cell *132*, 631–644.

Patro, R., Mount, S.M., and Kingsford, C. (2014). Sailfish enables alignment-free isoform quantification from RNA-seq reads using lightweight algorithms. Nat. Biotechnol. *32*, 462–464.

Patro, R., Duggal, G., Kingsford, C., 2015. Salmon: accurate, versatile and ultrafast quantification from RNA-seq data using lightweight-alignment. Published online June 27, 2015. http://dx.doi.org/10.1101/021592.

Pedregosa, F., Varoquaux, G., Gramfort, A., Michel, V., Thirion, B., Grisel, O., Blondel, M., Prettenhofer, P., Weiss, R., Dubourg, V., et al. (2011). Scikit-learn: machine learning in python. J. Mach. Learn. Res. *12*, 2825–2830.

Picelli, S., Björklund, Å.K., Faridani, O.R., Sagasser, S., Winberg, G., and Sandberg, R. (2013). Smart-seq2 for sensitive full-length transcriptome profiling in single cells. Nat. Methods *10*, 1096–1098.

Picelli, S., Faridani, O.R., Björklund, A.K., Winberg, G., Sagasser, S., and Sandberg, R. (2014). Full-length RNA-seq from single cells using Smart-seq2. Nat. Protoc. *9*, 171–181.

Pineault, N., Helgason, C.D., Lawrence, H.J., and Humphries, R.K. (2002). Differential expression of Hox, Meis1, and Pbx1 genes in primitive cells throughout murine hematopoietic ontogeny. Exp. Hematol. *30*, 49–57.

Poirault-Chassac, S., Six, E., Catelain, C., Lavergne, M., Villeval, J.-L., Vainchenker, W., and Lauret, E. (2010). Notch/Delta4 signaling inhibits human megakaryocytic terminal differentiation. Blood *116*, 5670–5678.

Reimand, J., Arak, T., and Vilo, J. (2011). g:Profiler–a web server for functional interpretation of gene lists (2011 update). Nucleic Acids Res. *39*, W307–W315.

Robin, C., Ottersbach, K., Boisset, J.-C., Oziemlak, A., and Dzierzak, E. (2011). CD41 is developmentally regulated and differentially expressed on mouse hematopoietic stem cells. Blood *117*, 5088–5091.

Sanjuan-Pla, A., Macaulay, I.C., Jensen, C.T., Woll, P.S., Luis, T.C., Mead, A., Moore, S., Carella, C., Matsuoka, S., Bouriez Jones, T., et al. (2013). Platelet-biased stem cells reside at the apex of the haematopoietic stem-cell hierarchy. Nature *502*, 232–236.

Schick, P.K., Konkle, B.A., He, X., and Thornton, R.D. (1993). P-selectin mRNA is expressed at a later phase of megakaryocyte maturation than mRNAs for von Willebrand factor and glycoprotein Ib-alpha. J. Lab. Clin. Med. *121*, 714–721.

Schulte, R., Wilson, N.K., Prick, J.C.M., Cossetti, C., Maj, M.K., Gottgens, B., and Kent, D.G. (2015). Index sorting resolves heterogeneous murine hematopoietic stem cell populations. Exp. Hematol. *43*, 803–811.

Seita, J., and Weissman, I.L. (2010). Hematopoietic stem cell: self-renewal versus differentiation. Wiley Interdiscip. Rev. Syst. Biol. Med. *2*, 640–653.

Song, H.-D., Sun, X.-J., Deng, M., Zhang, G.-W., Zhou, Y., Wu, X.-Y., Sheng, Y., Chen, Y., Ruan, Z., Jiang, C.-L., et al. (2004). Hematopoietic gene expression profile in zebrafish kidney marrow. Proc. Natl. Acad. Sci. USA *101*, 16240–16245.

Stachura, D.L., Reyes, J.R., Bartunek, P., Paw, B.H., Zon, L.I., and Traver, D. (2009). Zebrafish kidney stromal cell lines support multilineage hematopoiesis. Blood *114*, 279–289.

Tenen, D.G., Hromas, R., Licht, J.D., and Zhang, D.E. (1997). Transcription factors, normal myeloid development, and leukemia. Blood *90*, 489–519.

The GPy authors, n.d. GPy: A Gaussian process framework in python. http://github.com/SheffieldML/GPy.

Titsias, M.K., and Lawrence, N.D. 2010. Bayesian Gaussian process latent variable model. In Proceedings of the 13th International Conference on Artificial Intelligence and Statistics, pp. 844–851.

Van der Maaten, L., and Hinton, G. (2008). Visualizing data using t-SNE. J. Mach. Learn. Res. *9*, 85.

Vassen, L., Okayama, T., and Möröy, T. (2007). Gfi1b:green fluorescent protein knock-in mice reveal a dynamic expression pattern of Gfi1b during hematopoiesis that is largely complementary to Gfi1. Blood *109*, 2356–2364.

Wagner, G.P., Kin, K., and Lynch, V.J. (2012). Measurement of mRNA abundance using RNA-seq data: RPKM measure is inconsistent among samples. Theory Biosci. *131*, 281–285.

Wang, L., Wang, S., and Li, W. (2012). RSeQC: quality control of RNA-seq experiments. Bioinformatics *28*, 2184–2185.

Ward, J.H., Jr. (1963). Hierarchical grouping to optimize an objective function. J. Am. Stat. Assoc. *58*, 236–244.

Wright, D.E., Bowman, E.P., Wagers, A.J., Butcher, E.C., and Weissman, I.L. (2002). Hematopoietic stem cells are uniquely selective in their migratory response to chemokines. J. Exp. Med. *195*, 1145–1154.

Zeng, H., Yücel, R., Kosan, C., Klein-Hitpass, L., and Möröy, T. (2004). Transcription factor Gfi1 regulates self-renewal and engraftment of hematopoietic stem cells. EMBO J. *23*, 4116–4125.

Cell Reports

Supplemental Information

Single-Cell RNA-Sequencing Reveals a Continuous Spectrum of Differentiation in Hematopoietic Cells

Iain C. Macaulay, Valentine Svensson, Charlotte Labalette, Lauren Ferreira, Fiona Hamey, Thierry Voet, Sarah A. Teichmann, and Ana Cvejic

Supplemental Table legends

Table S1, Related to Figure 4. Top genes distinguishing each cluster based on random forest feature importance.

Table S2, Related to Figure 6. List of genes with dynamic expression over pseudotime, annotated by which temporal expression group they belong to. Together with enriched functional gene sets for the different temporal expression groups.

Table S3, Related to Figure 7. List of expressed Ohnolog gene pairs annotated by the decision tree classification into Single, Mixed and XOR -Ohnologs.

Supplemental Figure Legends

Figure S1. The gating strategy for sorting cd41-EGFP cells by flow cytometry, Related to Figure 1. First, debris was excluded by forward and side-scatter (A, D). Next, singlets were selected (B, E) and dead, PI positive cells, were excluded (C, F). Finally, autofluorescent cells were excluded from the analysis (G, H). The GFP positive population was split into GFP^{low} and GFP^{high} based on the level of GFP fluorescence (I).

Figure S2. Quality control assessment, Related to Figure 1. Quality control was assessed by analysing the number of detected genes compared to the number of input reads (A) or ERCC content (B). In each plate we sorted 94 cells, leaving two wells per plate without cells. Blue dots represent wells with cells and orange dots show wells without cells. Following sequencing and quality control, 13 cells were removed from further analysis. We excluded data points (cells) with few reads (less than 50,000) and few genes or with high ERCC content. As expected, wells without cells (orange) have ERCC content equivalent to 100%.

Figure S3. Pairwise plots of the four independent components used to represent the data, Related to Figure 2. A) The initial names of the components ("difference_component", "outlier_component", "within_large_component", "within_small_component") were given based on visual features. The dots, representing cells, are colored white for EGFP^{low} sorted cells and green for EGFP^{high} sorted cells. B) Ward clustering of the cells in ICA space. The clusters (here colored) were used to associate cells to progression along a component where the cluster varies the most.

Figure S4. The gating strategy for sorting cells from clusters 1a/1b/2, 3 and 4 by flow cytometry, Related to Figure 4. A-B) Plots of viable, single cells based on their GFP and PERCP fluorescence from either a non transgenic (A) or Tg(cd41:EGFP) (B) kidney single cell suspension. The GFP^{low} cells (C) can be further split into two groups based on SSC values: GFP^{low}SSC^{high} or GFP^{low}SSC^{low} (D). GFP fluorescence (E) and light scatter (F) properties of each cell coloured based on the cluster it belongs to. G) Stacked column graph showing the proportion of cells from each of the clusters in three different gates named here: GFP^{high}, GFP^{low}SSC^{low} and GFP^{low}SSC^{high}.

Figure S5. May-Grünwald Giemsa staining of cells from clusters 1a/1b/2, 3 and 4, Related to Figure 4. Cd41:EGFP cells were sorted based on GFP and SSC values to GFP^{low}SSC^{high},GFPl^{ow}SSC^{low} and GFP^{high}. Cytospin slides were prepared from sorted cells and stained with May-Grünwald Giemsa. The GFP^{low}SSC^{high} cells are enriched for cells from clusters 1a/1b/2, GFP^{low}SSC^{low} and GFP^{high} cells are enriched for cells from cluster 3 and 4 respectively.

Figure S6. Follow-up experiment, Related to Figure 5. A) Quality control of cells from the follow-up experiment. Out of 288 single cells, 19 were removed from further analysis due to having less than 200,000 sequenced reads, less than 150 detected genes or more than 99.5% ERCC spike-in content in the well. Thresholds were guided by control wells which were either empty or contained 50 cells. B) The data follow a similar pattern as in the original experiment (for comparison please see Figure S3A-B). Pairwise plots of three independent components representing the data from the follow-up experiment. The EarlyEnriched population is confined to the early progression along component 0 (corresponding to within_small_component in Figure S3B) before the switch in component 2 (corresponding to difference_component in Figure S3B). This corresponds to cluster 1a/1b/2 in the original data as expected. GFP^{high} cells from both the kidney and circulation completely overlap, indicating no further differentiation happens after the cells leave the kidney, and vary over component 1 (corresponding to within_large_component in Figure S3B).

Figure S7. The total mRNA content and number of expressed genes per cell are correlated with its differentiation state, not technical properties of the cells, Related to Figure 5. Light scatter properties FSC and SSC, total mRNA content, number of reads and the number of expressed genes in pseudotime. The dots, representing cells, are coloured based on the cluster the cells belong to.

Supplemental Data

Supplemental Data 1. Sample info, Related to Figures 1-7.

This table contains detailed information about each sample, which was inferred by analysis and used to create most figure panels.

Supplemental Data 2. Analysis files, Related to Figures 1-7.

This contains the scripts, in the form of IPython notebooks, to reproduce all analysis and most of the figure panels in the text.

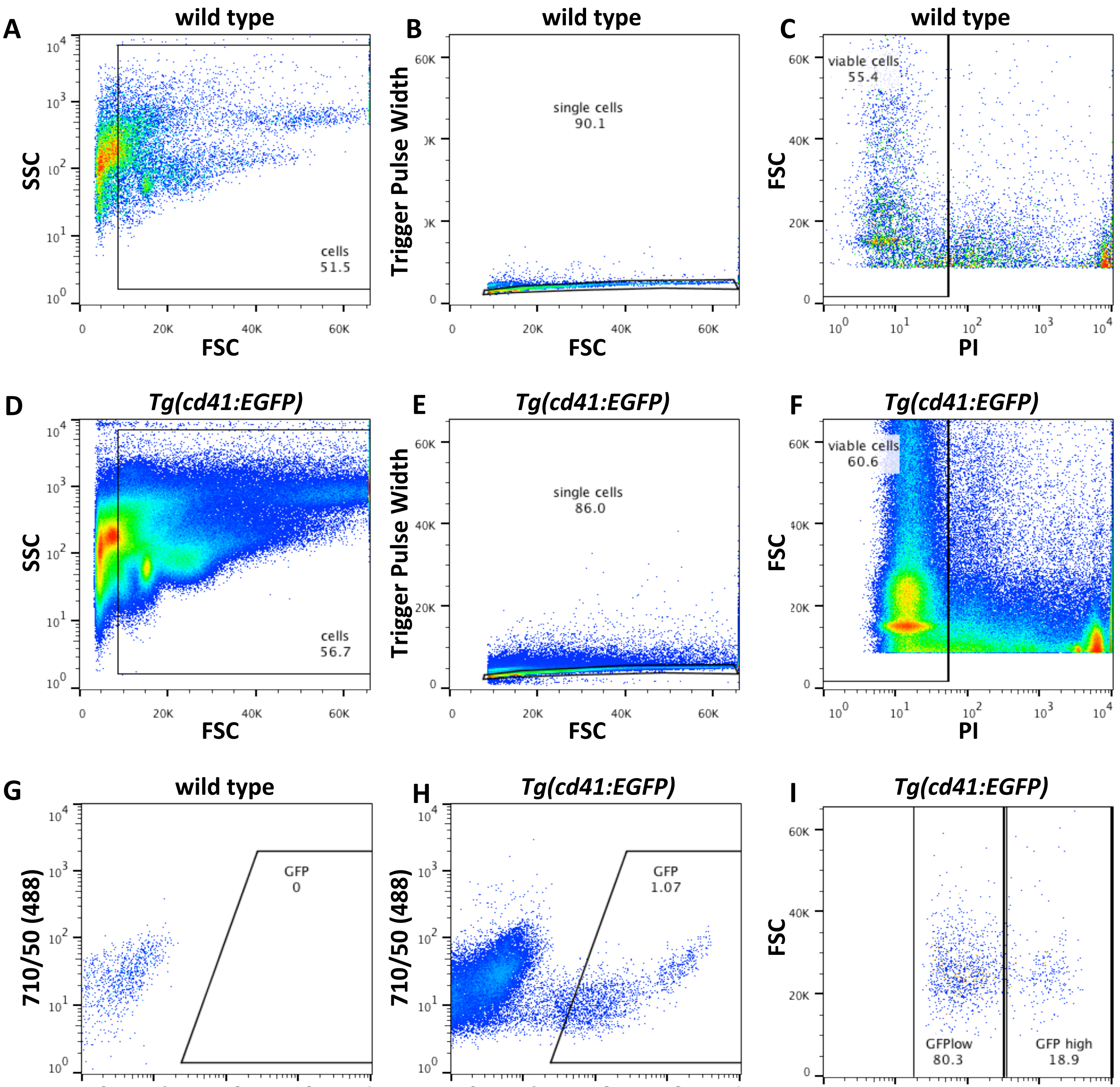
A
wild type
SSC
FSC
cells
51.5
B
wild type
Trigger Pulse Width
FSC
single cells
90.1
C
wild type
FSC
PI
viable cells
55.4
D
Tg(cd41:EGFP)
SSC
FSC
cells
56.7
E
Tg(cd41:EGFP)
Trigger Pulse Width
FSC
single cells
86.0
F
Tg(cd41:EGFP)
FSC
PI
viable cells
60.6
G
wild type
710/50 (488)
GFP
GFP
0
H
Tg(cd41:EGFP)
710/50 (488)
GFP
GFP
1.07
I
Tg(cd41:EGFP)
FSC
GFP
GFPlow
80.3
GFP high
18.9

Figure_S1

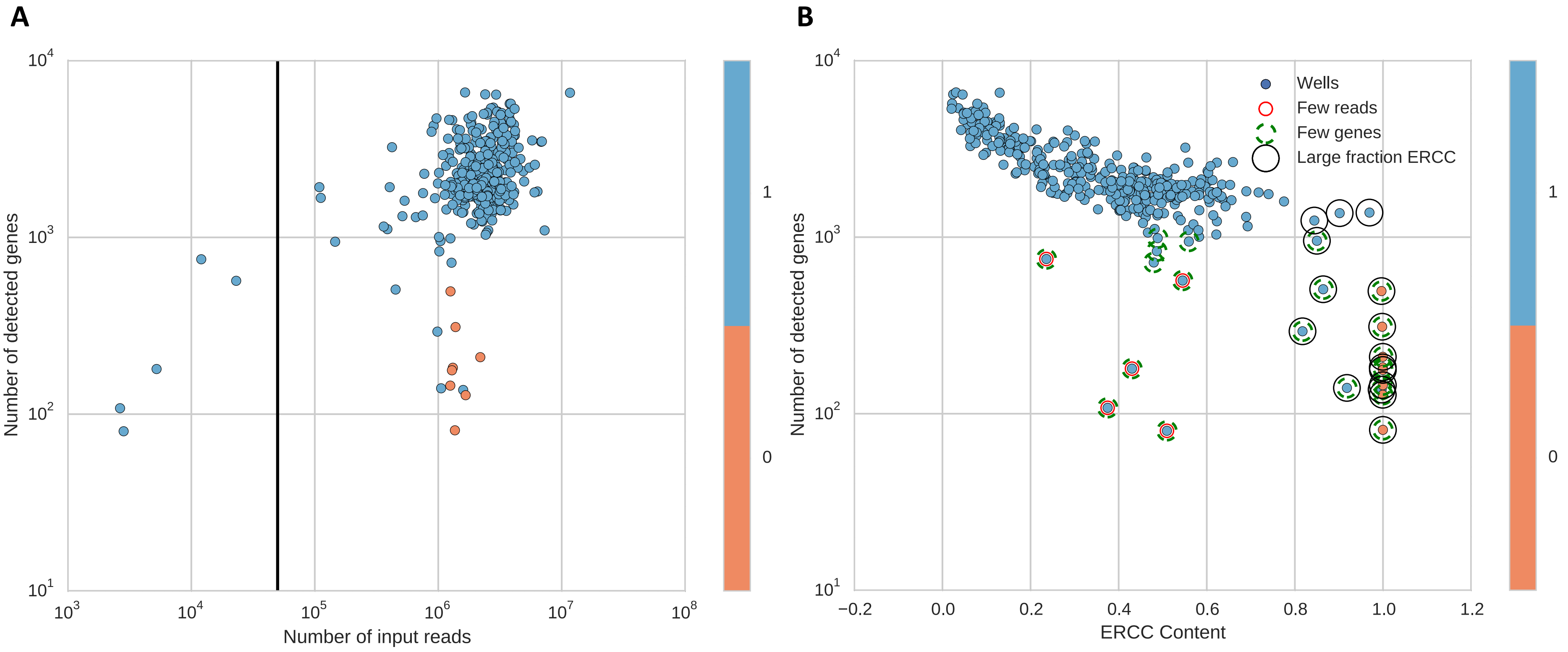
A
B
Number of detected genes
Number of input reads
ERCC Content
Wells
Few reads
Few genes
Large fraction ERCC
1
0

Figure_S2

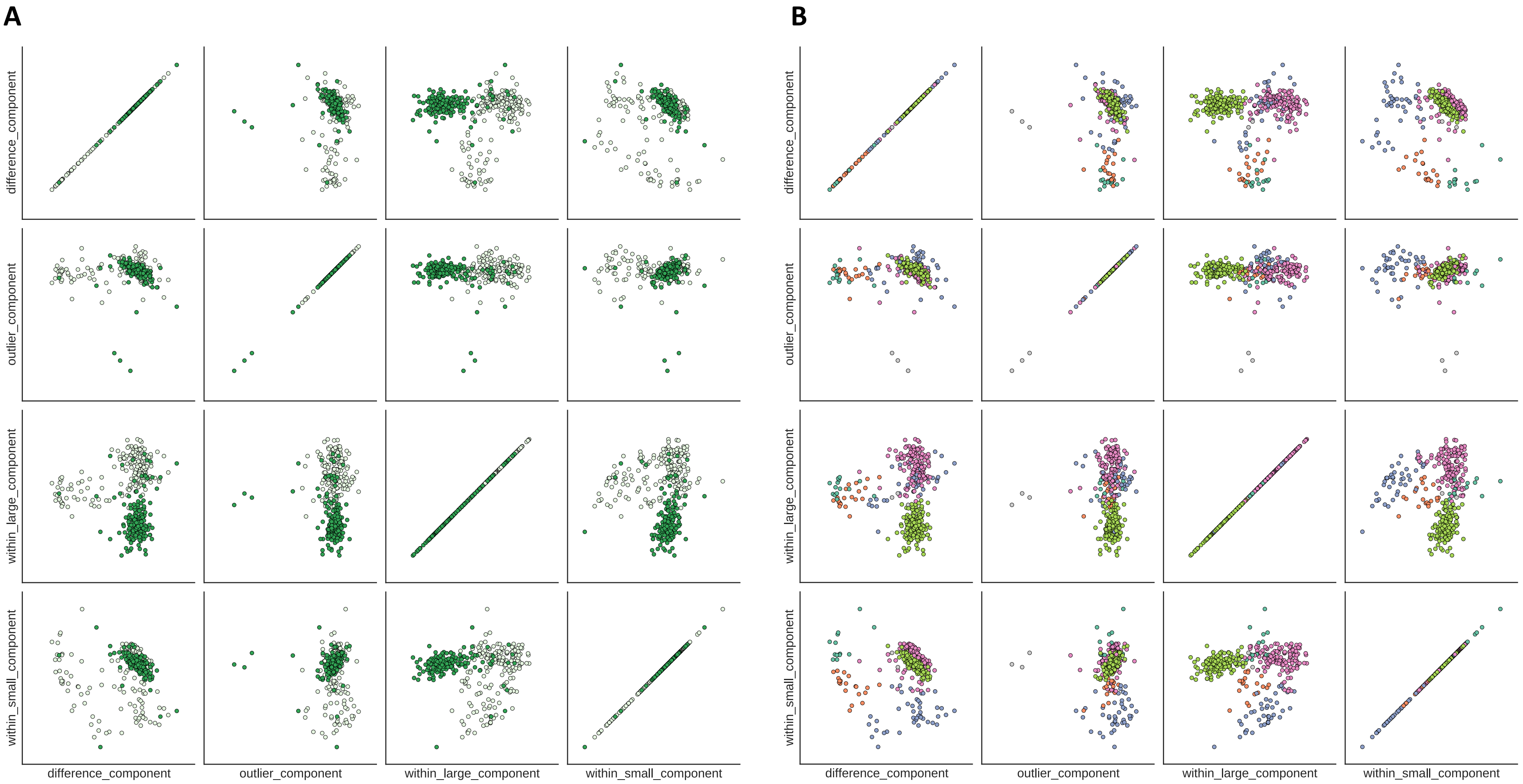
A
B
difference_component
outlier_component
within_large_component
within_small_component
difference_component
outlier_component
within_large_component
within_small_component
difference_component
outlier_component
within_large_component
within_small_component
difference_component
outlier_component
within_large_component
within_small_component

Figure_S3

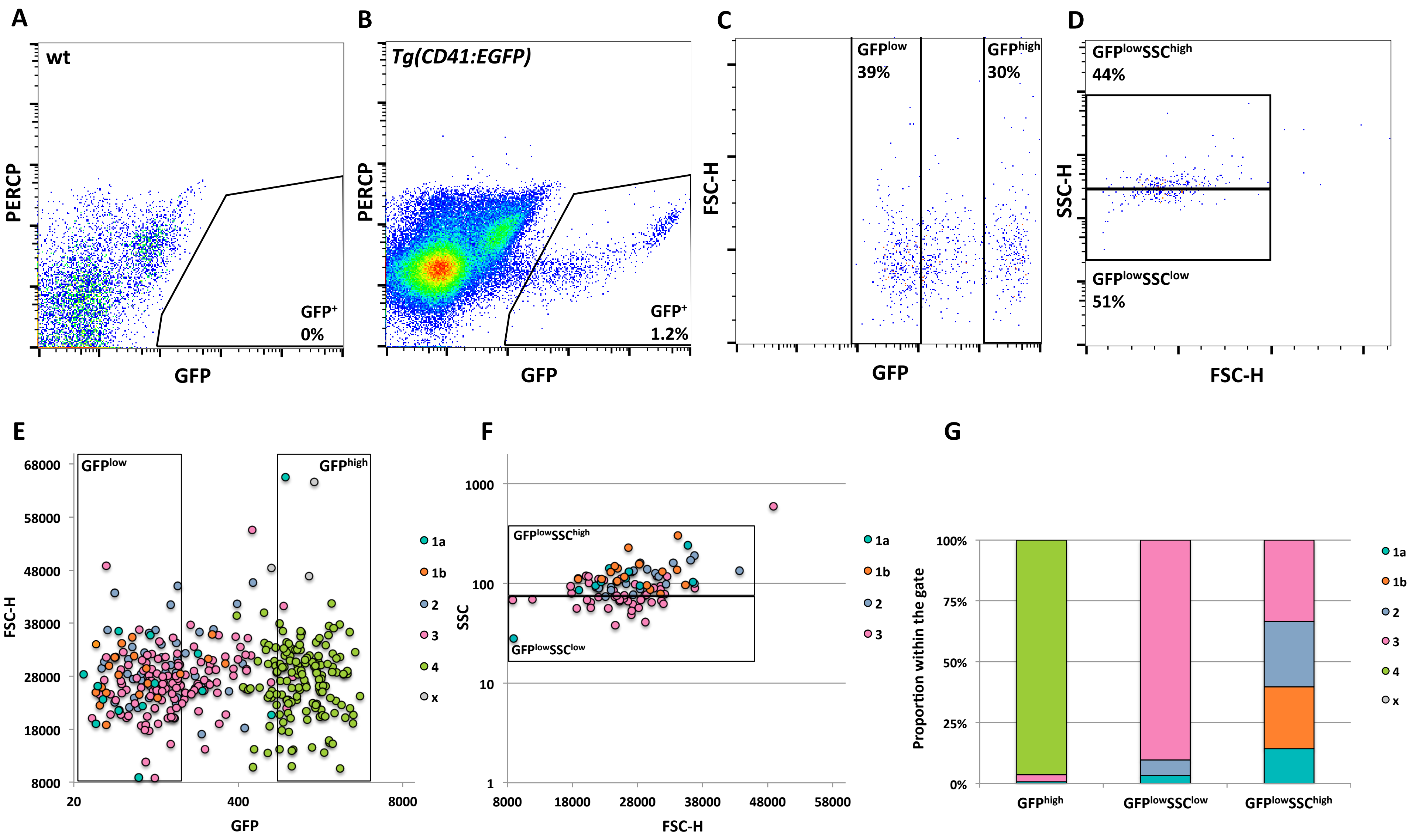
A
wt
PERCP
GFP
GFP+
0%
B
Tg(CD41:EGFP)
PERCP
GFP
GFP+
1.2%
C
GFPlow
39%
GFPhigh
30%
FSC-H
GFP
D
GFPlowSSChigh
44%
GFPlowSSClow
51%
SSC-H
FSC-H
E
GFPlow
GFPhigh
68000
58000
48000
38000
28000
18000
8000
FSC-H
20
400
8000
GFP
1a
1b
2
3
4
x
F
1000
100
10
1
SSC
GFPlowSSChigh
GFPlowSSClow
8000
18000
28000
38000
48000
58000
FSC-H
1a
1b
2
3
G
100%
75%
50%
25%
0%
Proportion within the gate
GFPhigh
GFPlowSSClow
GFPlowSSChigh
1a
1b
2
3
4
x

Figure_S4

GFP^{low}SSC^{high}
Clusters 1a/1b/2 enriched

GFP^{low}SSC^{low}
Cluster 3 enriched

GFP^{high}
Cluster 4

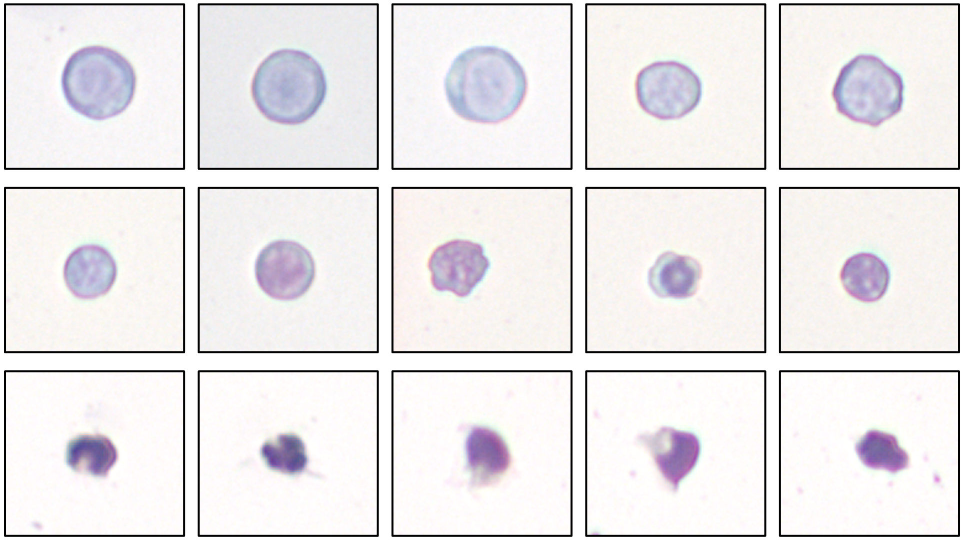

Figure_S5

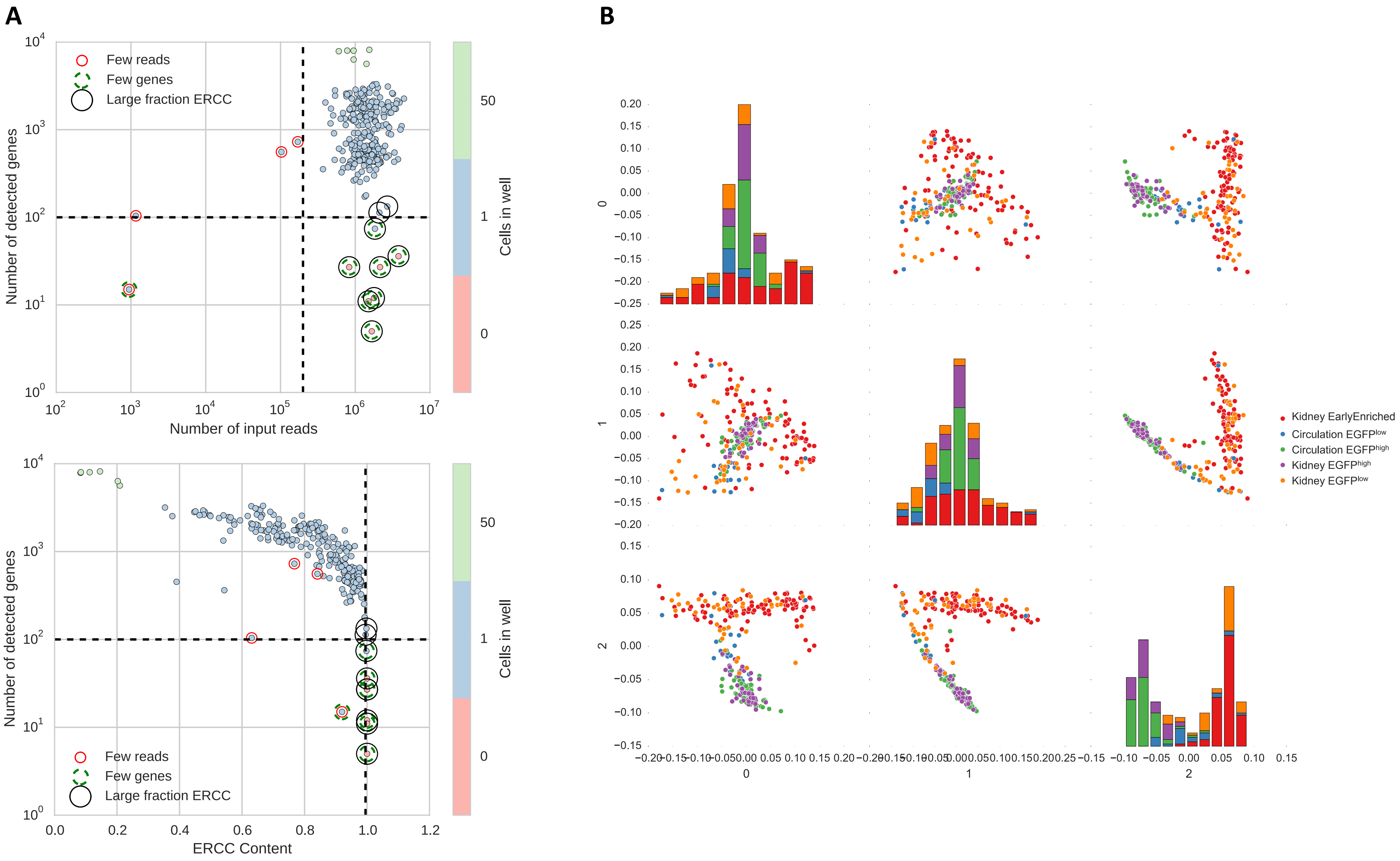
A
Few reads
Few genes
Large fraction ERCC
Number of detected genes
Number of input reads
Cells in well
ERCC Content
B
Kidney EarlyEnriched
Circulation EGFPlow
Circulation EGFPhigh
Kidney EGFPhigh
Kidney EGFPlow

Figure_S6

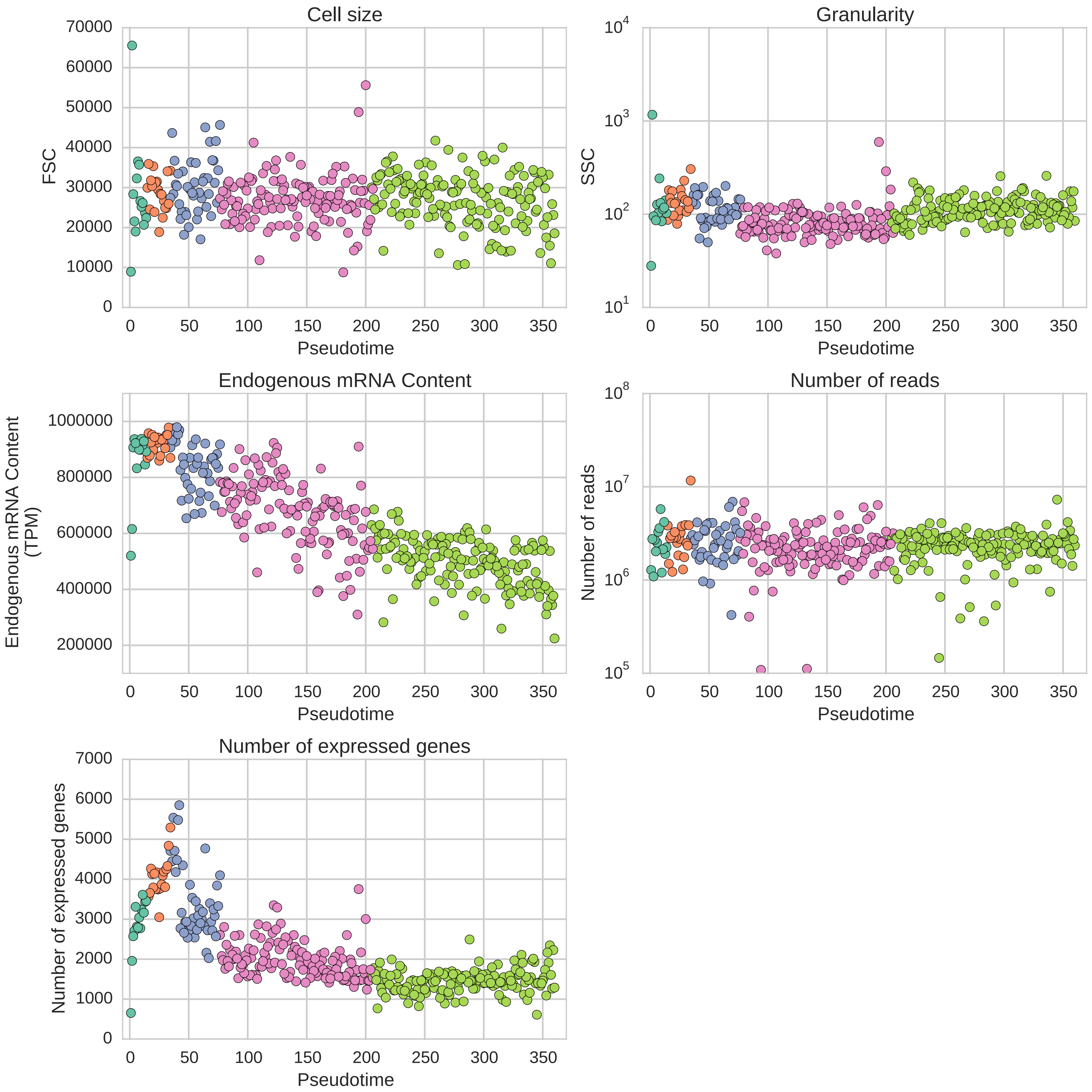
Cell size
FSC
Pseudotime
Granularity
SSC
Pseudotime
Endogenous mRNA Content
Endogenous mRNA Content (TPM)
Pseudotime
Number of reads
Number of reads
Pseudotime
Number of expressed genes
Number of expressed genes
Pseudotime

Figure_S7
